# A cell-based probabilistic approach unveils the concerted action of miRNAs

**DOI:** 10.1371/journal.pcbi.1007204

**Published:** 2019-12-02

**Authors:** Shelly Mahlab-Aviv, Nathan Linial, Michal Linial

**Affiliations:** 1 The Rachel and Selim Benin School of Computer Science and Engineering, The Hebrew University of Jerusalem, Jerusalem, Israel; 2 Department of Biological Chemistry, Institute of Life Sciences, The Hebrew University of Jerusalem, Jerusalem, Israel; Michigan State University, UNITED STATES

## Abstract

Mature microRNAs (miRNAs) regulate most human genes through direct base-pairing with mRNAs. We investigate the underlying principles of miRNA regulation in living cells. To this end, we overexpressed miRNAs in different cell types and measured the mRNA decay rate under a paradigm of a transcriptional arrest. Based on an exhaustive matrix of mRNA-miRNA binding probabilities, and parameters extracted from our experiments, we developed a computational framework that captures the cooperative action of miRNAs in living cells. The framework, called COMICS, simulates the stochastic binding events between miRNAs and mRNAs in cells. The input of COMICS is cell-specific profiles of mRNAs and miRNAs, and the outcome is the retention level of each mRNA at the end of 100,000 iterations. The results of COMICS from thousands of miRNA manipulations reveal gene sets that exhibit coordinated behavior with respect to all miRNAs (total of 248 families). We identified a small set of genes that are highly responsive to changes in the expression of almost any of the miRNAs. In contrast, about 20% of the tested genes remain insensitive to a broad range of miRNA manipulations. The set of insensitive genes is strongly enriched with genes that belong to the translation machinery. These trends are shared by different cell types. We conclude that the stochastic nature of miRNAs reveals unexpected robustness of gene expression in living cells. By applying a systematic probabilistic approach some key design principles of cell states are revealed, emphasizing in particular, the immunity of the translational machinery vis-a-vis miRNA manipulations across cell types. We propose COMICS as a valuable platform for assessing the outcome of miRNA regulation of cells in health and disease.

## Author summary

Alteration in miRNA expression occurs throughout cell differentiation, inflammation, viral infection, tumorigenesis, and other pathologies. Notwithstanding a rich body of experimental data intended to assess the outcome of miRNA alterations in cells, the underlying design principles remain obscure and fragmented. In this study, we develop a quantitative stochastic model that simulates the mRNA steady-state in view of alteration in miRNAs’ abundance. We systematically analyzed the behavior of miRNA-mRNA regulation and confirm that the stochastic nature of miRNA regulation reveals unexpected robustness of cell behavior across cell types. Specifically, we expose the immunity of the translational machinery towards miRNA regulation. The developed platform, called COMICS compares the results of miRNA regulation across various cell types. Based on stochastic and probabilistic considerations, we provide a dynamic and flexible framework that quantifies the competition of miRNAs within cells in health and disease.

## Introduction

Mature microRNAs (miRNAs) are small, non-coding RNA molecules (∼22 nucleotides) that regulate genes through base-pairing with their cognate mRNAs, mostly at the 3′ untranslated region (3’-UTR) [1–3]. In multicellular organisms, miRNAs act post-transcriptionally by affecting the destabilization and degradation of mRNAs, as well as interfering with the translation machinery [4–6]. Switching between cell states is accompanied by a shift in the profile of miRNAs [7]. Indeed, miRNA-dependent transitions are documented in cells undergo quiescence [8], differentiation [9], viral infection [10] and cancer transformation [11, 12].

In humans, there are ~2500 mature miRNAs that derive from ~1900 genes [1]. Studies of miRNA-mRNA regulatory networks reveal that almost all coding genes have multiple putative miRNA binding sites (MBS) at their 3’-UTR [13–15], and many miRNAs can possibly target hundreds of transcripts [16, 17]. However, current estimates postulate that only ~60% of the human coding genes are regulated by miRNAs [18, 19]. Most of our knowledge of the specificity of miRNA-mRNA network is based on computational prediction tools [20] that use parameters learned from in-vitro overexpression or miRNA knockdown experiments [21]. Additionally, the CLIP-Seq experiments produce bulk lists of interacting miRNAs and mRNAs from healthy and diseased cells [22]. A revised protocol, called CLASH, provides validated pairs of miRNAs and their binding site sequences (MBS) on targeted mRNAs [2, 23]. Unfortunately, many of the above protocols suffer from low coverage and poor consistency (discussed in [24]).

A quantitative perspective for miRNA regulation is strongly dependent on the identity and quantity of limiting factors in living cells. Example is the AGO protein, a crucial catalytic component of the RNA silencing complex (RISC) [25, 26]. From the mRNA perspective, the number of miRNA molecules, and the positions of MBS along the relevant transcript determine the potential of miRNA interactions [27]. The outcome is a rich regulatory network displaying a “many to many” relation of miRNAs and mRNAs. Such design supports noise reduction [28–30], and robustness against environmental fluctuation [31].

A cellular view of miRNAs networks was formulated by the ceRNA hypothesis [32, 33]. Accordingly, overexpression of MBS-rich molecules of RNA may displace miRNAs from their primary authentic targets [34, 35], resulting in an attenuation relief of specific mRNAs. The result of such a competition is an interplay between direct and indirect effects on gene expression [36, 37]. The dynamics of the miRNA-target regulatory network in view of direct and distal regulation had been modeled [38]. It was further postulated that many of the miRNA weak sites contribute to target-site competition without imparting repression [34].

In this paper, we describe a quantitative stochastic model that challenges the cell steady-state in view of alteration in miRNAs’ abundance. Our model operates at the cellular level and compares the overall trend of miRNA regulation in various human cell lines. We have systematically analyzed the behavior of miRNA-mRNA interacting pairs. This analysis confirmed that the stochastic nature of miRNA regulation reveals unexpected robustness of the translational machinery in living cells.

## Results

### Determining miRNAs stability and decay rate of mRNAs upon transcriptional arrest

The nature and extent of miRNA regulation in living cells are depicted by the absolute quantities, composition, and stoichiometry of the miRNAs and mRNAs [39]. In this study, we model the outcome of the miRNA-mRNA network under a simplified paradigm in which the synthesis of new transcripts (miRNA and mRNAs) is prevented.

**Fig 1:**
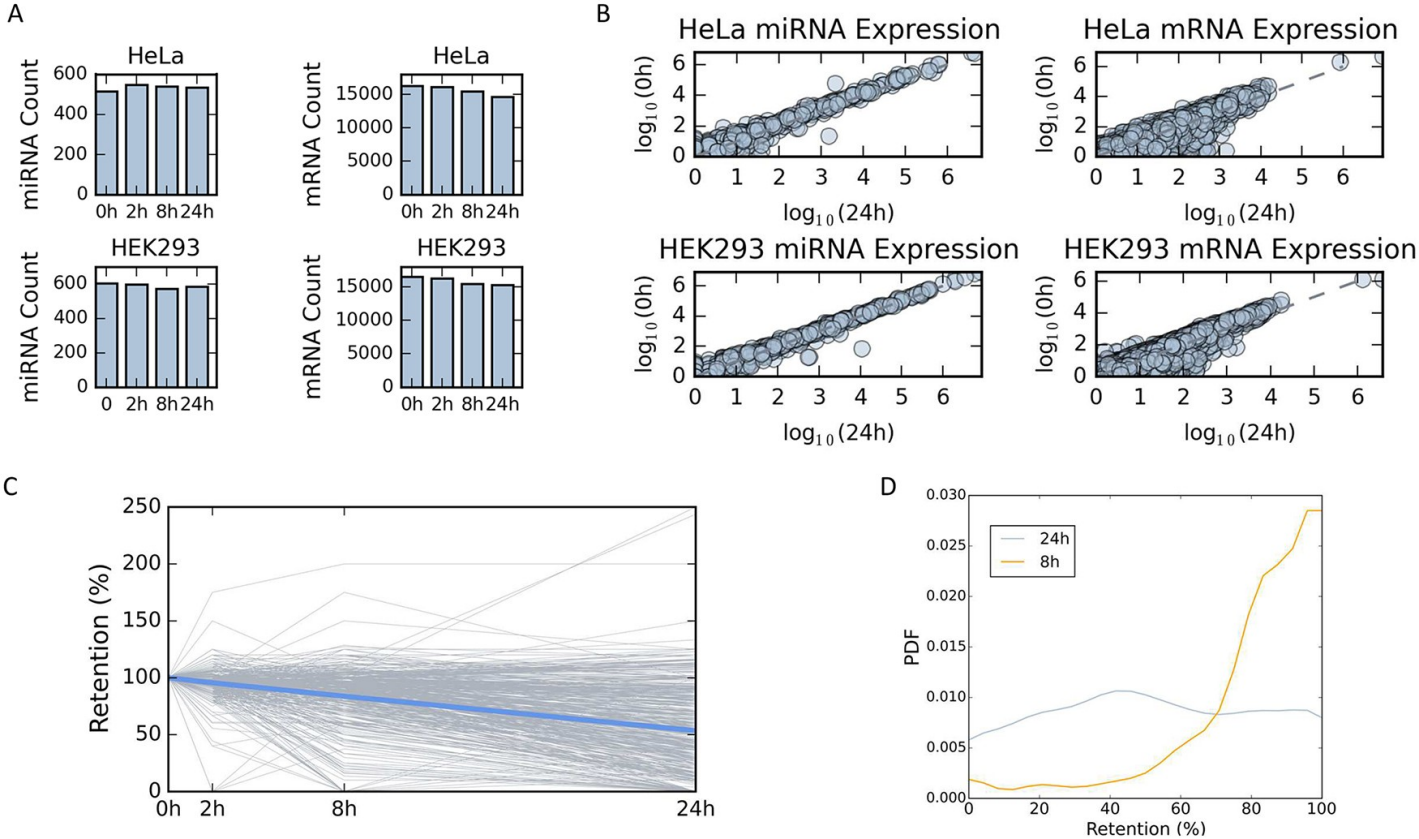
Expression profiles of miRNA and mRNA under transcription arrest. Counting miRNAs (left) and mRNAs (right) for the 4 different time points for HeLa (top) and HEK-293 (bottom). **(A)** The samples were collected at 0, 2, 8, and 24 hrs following transcription inhibition by ActD. **(B)** Expression of miRNAs (left) and mRNAs (right) in pairs of 4 different time points for HeLa (top) and HEK-293 (bottom). Gene expression is presented by logarithmic scale (log_10_). Spearman correlation (r) is listed for each pair along with the p-value of the significance. Source data is available in S1 Dataset (HeLa) and S2 Dataset (HEK-293). **(C)** Relative abundance of each expressed mRNAs for HeLa cells. At time 0, the relative abundance is set to 100%, and at each proceeding time points the abundance relative to time 0 is reported. Each line represents a single gene (mRNA). Only genes with a minimal expression level of 0.02% expression are listed (equivalent to 97 FPKM, total of 860 genes). The blue line represents the average of all reported genes at each time point. **(D)** Compilation of mRNA retention distribution (probability density function, PDF) of all the reported genes after 8 hrs and 24 hrs from initiation of transcription inhibition by ActD. All genes with a retention level ≥100 are combined (at 100% retention).

We first tested the relative changes in the quantities of miRNAs and mRNAs in HeLa and HEK-293 cell-lines, in the presence of the transcriptional inhibitor Actinomycin D (ActD, Fig 1A). Overall, we mapped 539 and 594 different miRNAs in untreated HeLa and HEK-293 cells, respectively (Fig 1A). In addition, 16,236 and 16,463 different expressed mRNAs (not including miRNAs) were mapped from HeLa (S1 Dataset) and HEK-293 cells (S2 Dataset), prior to ActD treatment, respectively. We then tested the composition of miRNAs and mRNAs 24 hrs post-treatment. Importantly, the number of miRNA molecules 24 hrs after the application of the drug remains constant in HeLa (Spearman rank correlation, r = 0.94) and HEK-293 cells (Spearman rank correlation, r = 0.97, Fig 1B). In contrast, the number of mRNAs molecules has monotonically declined in accordance with the effect of ActD on the bulk of mRNAs (Fig 1A). Maximal variability in the profile of mRNAs is measured between 0 hr and 24 hrs for HeLa (Fig 1B, Spearman rank correlation, r = 0.84, top right) and HEK-293 cells (Spearman rank correlation, r = 0.88, bottom right, right). Supporting figures show the pairs for all other time points for HeLa (S1 Fig) and HEK-293 (S2 Fig), respectively.

Fig 1C follows the changes of the expression of individual genes in HeLa cells along 24 hrs following the drug treatment. A change in each mRNA abundance is quantified relative to its expression level at the starting point. To avoid numerical instability, we only report on the retention percentage for genes that are expressed above a predetermined threshold (see Materials and methods, a total of 860 genes, Fig 1C). We illustrate how the overall distribution of the retention level (in %, 860 genes) varies between two time points, 8 hrs and 24 hrs (Fig 1D). The average retention rate is ~83% after 8 hrs and decreases to 53% 24 hrs following ActD treatment. These results validate that the decay rate for most mRNAs is a gradual process that continues for 24 hrs.

### Direct targets and non-target mRNAs are affected by miRNAs overexpression

**Fig 2:**
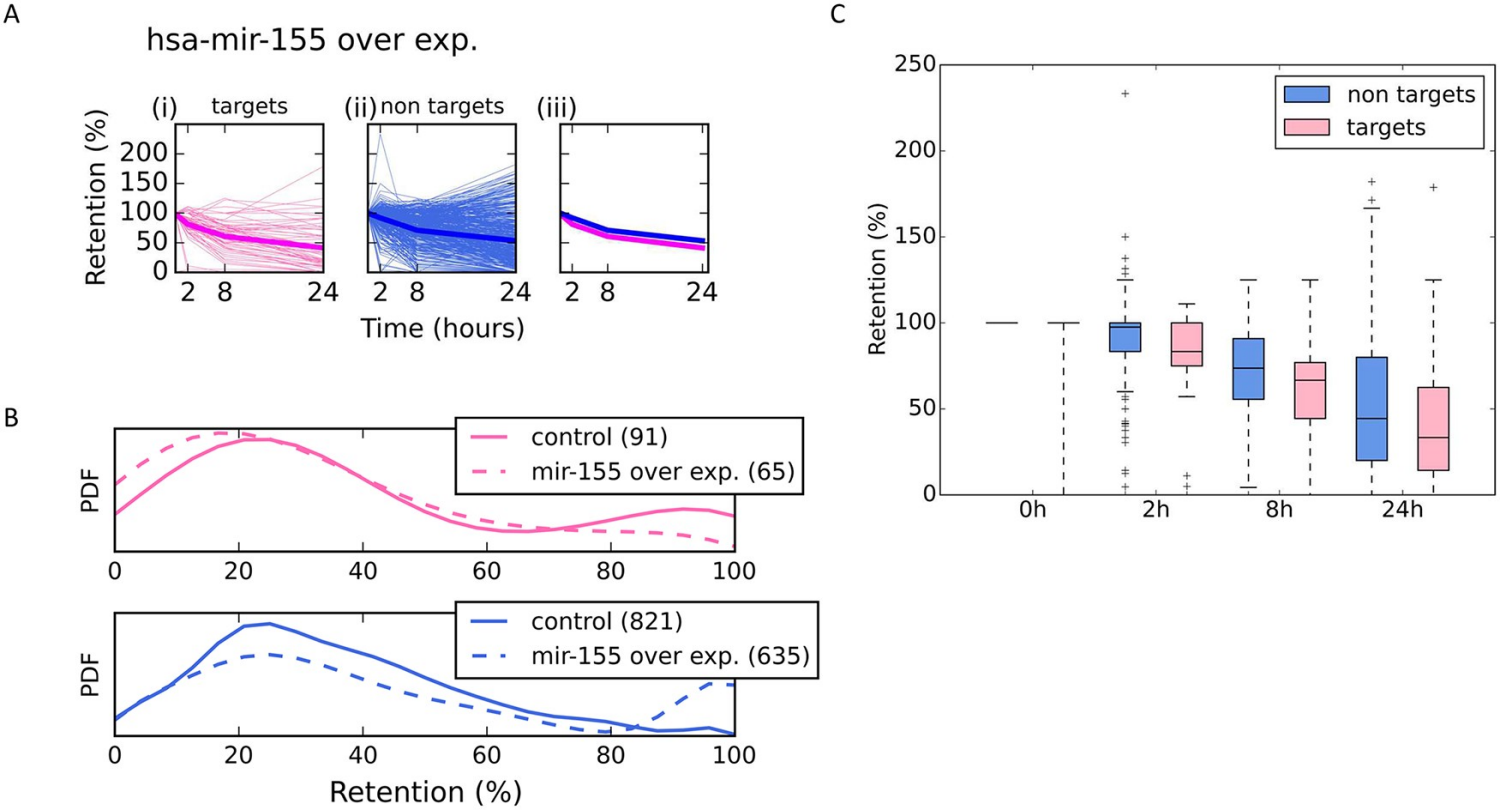
Retention profile of mRNAs following overexpressing miRNAs in HeLa cells. Relative mRNA retention in HeLa cells that were transfected and overexpressed with hsa-mir-155. Measurement were taken at 4 time points as indicated. **(A)** The retention plots are partitioned to target genes (i) (pink, left panels) and (ii) non-target genes (blue, middle panels). In (iii), the pink and blue lines are the average retention patterns for hsa-mir-155 for the targets and non-targets, respectively. **(B)** Distribution of genes retention after 24 hrs following the ActD treatment, according to their labels as targets (upper panel, pink) and non-targets (lower panel, blue). The plots compare the retention of genes from the control (smooth line), and from hsa-mir-155 overexpressed condition (dashed line). The number of genes that are included in the analyses are indicated in parentheses. Target genes are marked by pink lines (top) and the non-target genes by blue lines (bottom). Note the shift in the distribution in the non-target genes towards the genes with higher retention level. All genes with a retention level ≥100 are shown as 100% retention. **(C)** Retention levels are summarized by the box plots showing all time points following the ActD treatment (0 hr, 2 hrs, 8 hrs and 24 hrs) for target (pink) and non-target (blue) gene lists.

Fig 2 shows the results of direct and indirect effects of overexpressing hsa-mir-155. HeLa cells were transfected with individual miRNAs, and the number of miRNA and mRNA molecules was measured 24 hrs after the addition of ActD. We quantified the effect of hsa-mir-155 by considering its predicted targets. Specifically, for each miRNA, we split all expressed genes into targets and non-targets gene sets, according to TargetScan 7.1 table [27] (see Materials and methods). Retention rates of all genes relative to their starting point are shown (Fig 2A). The average decay rate for hsa-mir-155 direct targets is slightly faster compared with the larger set of non-target genes (Fig 2A, compare pink and blue thick lines). Furthermore, the decay after 24 hrs from ActD treatment for HeLa cells overexpressing hsa-mir-155 is enhanced in the transfected vs. naïve cells (Fig 2B, upper panel). While the shift in the relative mean statistics (Fig 2B) for the direct targets is marginal (p-value = 0.122), the shift for the non-target genes is significant (p-value of 0.002, Fig 2B, compare solid and dashed lines). A boxplot summary of all time points partitioned to target genes and non-target gene sets is shown (Fig 2C). Repeating the test for all time points confirmed the trend for each of the experimental time points (0 hr = 1.72e-03, 2 hrs = 3.36e-05, 8 hrs = 3.73e-03 and 24 hrs = 2.47e-2). The result implies a certain degree of stabilization for the non-target genes as a result of hsa-mir-155 overexpression. A similar trend for the retention profile from HeLa cells overexpressing hsa-mir-124a was observed (S3 Fig).

We conclude that under the described experimental settings, the miRNA regulatory network affects the probabilities of miRNA-target interactions, mostly by an indirect propagation of interactions, presumably due to competition on MBS, along with a continuous change in the miRNAs-mRNAs stoichiometry.

### A probabilistic approach for miRNA—mRNA interactions

The experimental results (Figs 1 and 2) emphasize the need for a systematic approach for studying miRNA-mRNA interaction network while considering the quantitative constraints in living cells. For our computational approach, we designed a stochastic process in which miRNAs and mRNAs compete dynamically, where the miRNA-mRNA binding probabilities dictate the level of suppression of gene expression. We used the miRNA-MBS interaction matrix from TargetScan, where each interaction is associated with a probability score. These scores are a proxy for the effectiveness of miRNA binding to a specific MBS. Genes that lack MBS in their 3’-UTR, are not listed in the TargetScan matrix and are excluded from our analysis. Altogether, TargetScan matrix reports on over 8.2 M interactions many of which are questionable. Rather, we restrict the analysis to the 1,183,166 interactions which are annotated by TargetScan as high-quality miRNA-mRNA pairs (see Materials and methods, and S1 Text).

**Fig 3:**
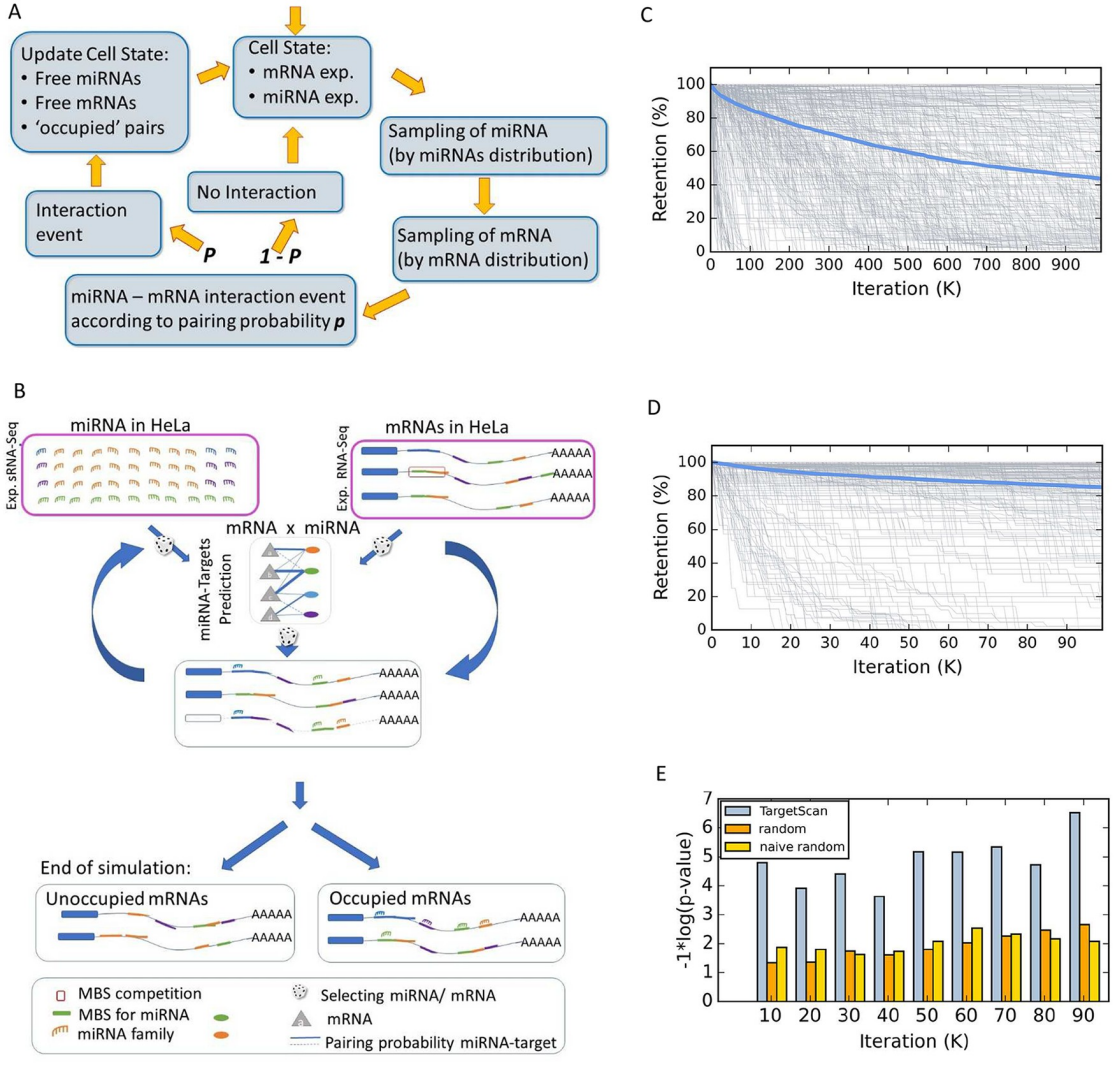
Scheme of the COMICS platform and performance of the simulation process. A schematic view for a single iteration of COMICS simulation. **(A)** A scheme for a single iteration step. After each successful interaction step, the distributions of the miRNA and mRNA in the cells are updated. Therefore, the next iteration is slightly changed due to resetting of the mRNA composition and availability of the free pool of miRNAs. The input for the simulator matches the experimentally determined molecular profile of the cell under study. **(B)** The outline of the major steps of COMICS operation from the mRNA perspective. The composition of miRNAs in the cells is obtained from the experimental measurement at 0 hr, normalized for 50k miRNAs and of 25k mRNAs. For HeLa cells, these are 3666 types of mRNAs that are included in the analysis (i.e. above a minimal threshold). Sampling of the miRNA and mRNAs is done according to their distribution and the probability of the interaction according to TargetScan interaction matrix (with 1.2 M values). A dashed mRNA shown after N iterations signifies an occupied transcript that is still halted prior to its removal form the list, and releasing of its bound miRNAs. **(C)** The retention of HeLa expressed genes along COMICS simulation for for 1M iterations and 100 k iterations **(D)**. COMICS simulation on input from HeLa cells reports on 3666 types of mRNAs and 110 of miRNAs. These numbers account for the 50k and 25k molecules of miRNAs and mRNAs, respectively. Each grey line represents the retention profile of a single type on mRNA. The blue line shows the mean retention. For graphical clarity, only mRNA above a predefined expression (>0.02%) are shown. **(E)** Testing COMICS performance and dependency on the information in TargetScan interaction matrix. COMICS simulation performance in HEK-293 was compared to experimentally validated pairs as reported from CLASH data on HEK-293. The histogram shows the performance in term of the significant of the overlap of the reported COMICS results (for each 10k, total 100k iterations) using TargetScan probabilistic converted matrix (grey), and two versions of randomization for the interaction table (S7 Fig). The statistical test was based on the 251 genes that are reported as pairs miRNA-mRNA pairs by CLASH and expressed above the minimal expression threshold used for COMICS simulation protocol. The use of the TargetScan matrix shows significant results versus CLASH data (at the significant range p-value of 1e-4 to 1e-6). Applying any of the randomizations for the miRNA-MBS interaction table, caused a drop in the performance to non-significant values.

We set to investigate the properties of the miRNA-mRNA interaction network in living cells. To this end, we developed an iterative simulator called COMICS (COmpetition of MiRNAs Interactions in Cellular Systems). Fig 3A illustrates a single iterative cycle of COMICS. The probabilistic framework relies on a constant updating of the cell-state. The cell state is sensitive to the actual quantities of occupied versus free molecules, and to the calculated miRNAs and mRNAs distributions. COMICS iterations are designed to capture the stochastic nature of miRNA regulation in living cells.

Fig 3B is a breakdown of the COMICS process through the lens of the targeted mRNAs. Importantly, while the nature and the positions of MBS at the 3’-UTR of a transcript are given for each mRNA variant, the expression profiles of miRNAs and mRNAs differ across cell types (S1 Dataset (HeLa), and S2 Dataset (HEK-293)). Therefore, the composition of miRNAs and mRNAs, and their absolute numbers in a cell dominate the sampling process (Fig 3B, pink frames). At each iteration, a miRNA is randomly sampled, with probability proportionate to its relative abundance. Next, one of its target genes is randomly chosen according to the measured expressed mRNAs distribution. A binding event may occur according to the binding probability of the sampled miRNA-mRNA pair. Following such event, the distributions of both, the miRNA and mRNA are updated (Fig 3A and 3B). Specifically, the status of the mRNA following a successful pairing change accordingly (i.e., marked as ‘prone to degradation’). The status of mRNA as ‘occupied’ and ‘prone to degradation’ does not prevent it from engaging in subsequent bindings. However, binding to MBS in close proximity to an occupied site on the same transcript is excluded. Such overlapping MBS are defined according to a minimal spacing between them. An occupied mRNA is marked for degradation with some delay, mimicking the in-vivo likely scenario of multiple miRNA binding on the same transcript. The cellular experiments (Fig 1B, S1 and S2 Figs) confirmed that miRNAs are extremely stable and their level is unchanged. Thus, once an occupied mRNA is removed, all bounded miRNAs return to the free miRNA pool. As a result, the stoichiometry of miRNA to mRNA is gradually changing with an increase in the ratio of miRNAs to free mRNAs.

The results of COMICS run for 100k and one million iterations on HeLa cells are shown. Fig 3C and 3D show the decay rate of 755 genes whose expression exceeds a predetermined threshold (>0.02% of mRNA molecules). We observed that changes in gene expression (measured by the retention level) are most pronounced at the initial phase of the COMICS run (i.e. 100k iterations). Following 1M iterations, the mean retention of mRNAs continues to drop (to 43.5%, 1M iterations, Fig 3C). A similar degree of decay was observed following 24 hrs of transcriptional arrest in living cells (Fig 2). The S3 Dataset reports on the output of mRNA expression as produced by COMICS, along the 1M iteration run. Comparing the results from COMICS with the cell experiments following 24 hrs of ActD on Hela and HEK-293 cells showed high correlation at 100k (Spearman correlation of 0.6 and 0.58, respectively). The correlation dropped to 0.38 (HeLa) and 0.35 (HEK-293) at 1M iterations (S4A Fig). The rest of the analyses are based on COMICS run with 100k iterations. It is consistent with the more sensitive phase of the simulation, as illustrated by comparing the changes in retention of mRNAs relative to the beginning of the simulations (p-value of Wilcoxon signed-rank test = 1e-94, S4B Fig).

The output of COMICS and results from a direct miRNA-mRNA pairing experiment were compared (Fig 3E). Specifically, we tested the correspondence along the COMICS run with respect to results from the CLASH experiment [23] (Fig 3E), both performed on HEK-293 cells. The overlap of our data and the CLASH [23] experiment is highly significant throughout the iteration run (Fig 3E, hypergeometric test p-value = 0.0014). Remarkably, replacing the TargetScan miRNA-MBS interaction matrix with two versions of randomized tables (see Materials and methods, and S1 Text) eliminated the statistical agreement between CLASH and COMICS results (Fig 3E). We conclude that the stochastic probabilistic protocol used by COMICS faithfully simulates global trends in miRNA regulation that takes place in living cells.

### Simulating miRNA overexpression by COMICS reveals stabilization of non-target genes

**Fig 4:**
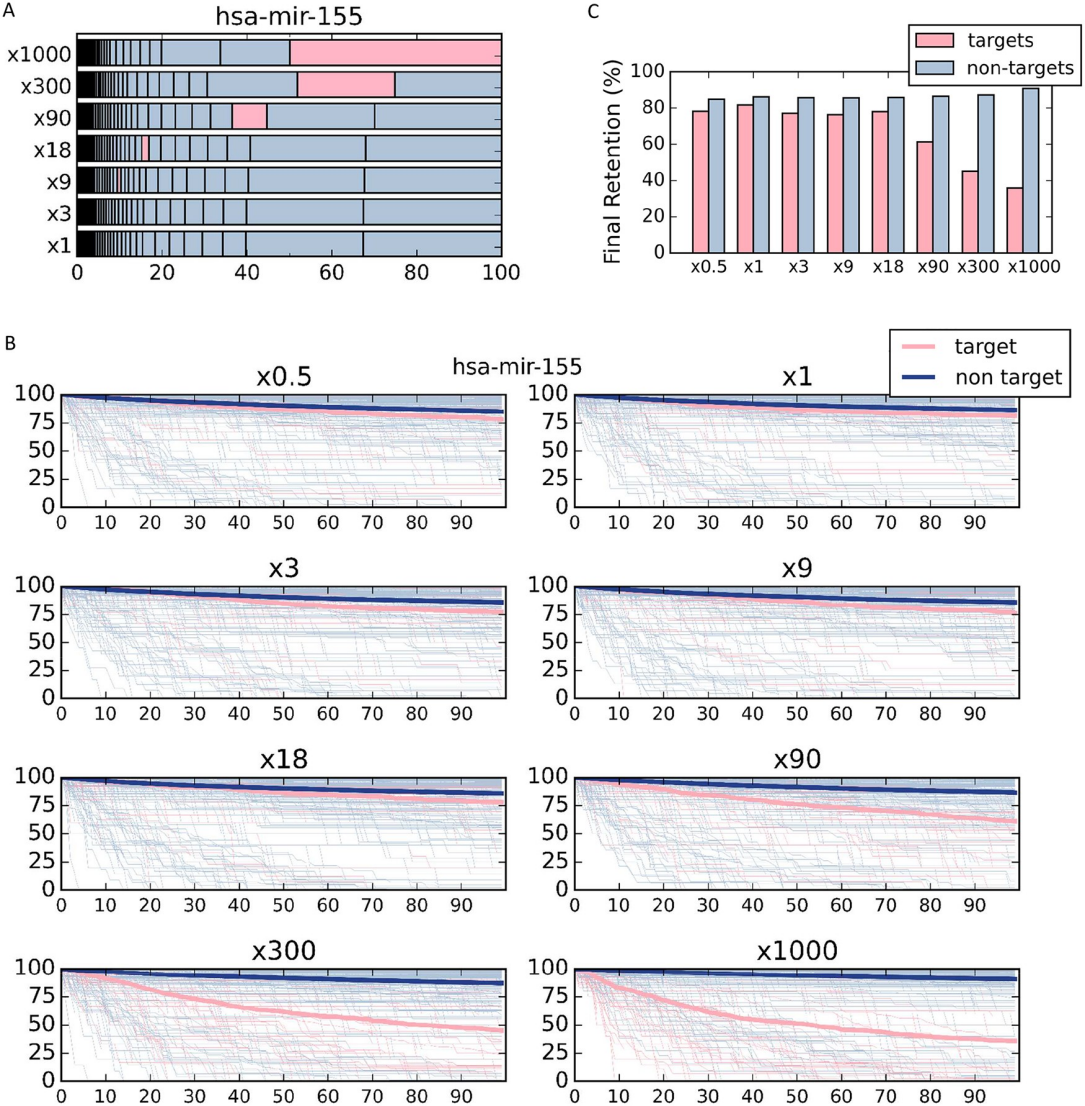
miRNA overexpression paradigm using COMICS platform. **(A)** The percentage of miRNA is sorted from lowest (left) to highest (right). Seven different hsa-mir-155 over expression simulations are shown (x1, x3, x9, x18, x90, x300 and x1000) from bottom to top. The fraction of hsa-mir-155 from the entire miRNA pool in the system is marked in pink. The relative percentage of each miRNA abundance is separated by vertical line. **(B)** Overexpression simulation of hsa-mir-155 in eight different overexpressing levels (marked by multiplication factors), where hsa-mir-155 target genes are marked in pink and all other non-target genes are marked in gray. The mean retentions of both gene groups are plotted in bold lines. **(C)** Average final retention of the different simulation runs, using different overexpression factors of hsa-mir-155 as shown in B partitioned to hsa-mir-155 target and non-target genes. A statistical test was performed to test for the significance of the final retention for hsa-mir-155 x1 and x1000. The t-test p-value for the target genes is 5.77e-12 and for the non-target genes is 0.00054.

COMICS makes it possible to perform exhaustive overexpression experiments while testing the impact on mRNA decay. Notably, miRNA profiles are among the strongest characteristics of cell identity and cell states. We activated COMICS by manipulating the abundance of hsa-mir-155 from its native state (x1, no overexpression) to varying degrees of overexpression (marked by multiplication factors: x0.5, x3, x9, x18, x90, x300 and x1000) using the probabilistic in-silico simulation framework (Fig 3B). In practical terms, overexpression of a single miRNA causes the re-distribution of all other expressed miRNAs (Fig 4A). Fig 4A illustrates that overexpression of miRNA (hsa-mir-155) by a factor of x300 and x1000. Under these conditions, hsa-mir-155 comprises 20% and 50% of all miRNAs respectively. It is important to note that the calculated fraction of each miRNA in the cell following overexpression depends on its initial abundance in the native cell.

Fig 4B demonstrates the gradual change in mRNA retention of each gene (above a predetermined threshold) along the 100k iterations of COMICS simulation (source data in S4 Dataset for HeLa and HEK-293 cells). The analysis reveals that the final retention level is sensitive to the degree of overexpression (Fig 4C). In the case of hsa-mir-155 in HeLa cells, elevating the miRNA from x18 to x90 caused a drop in the average retention of its targets, a trend that is even more prominent at higher overexpression levels. A minor, but consistent and significant increase in the retention of non-target genes (Fig 4C, gray color) is observed (t-test p-value = 0.00054).

### A unified pattern of mRNA retention is associated with overexpression of miRNAs

**Fig 5:**
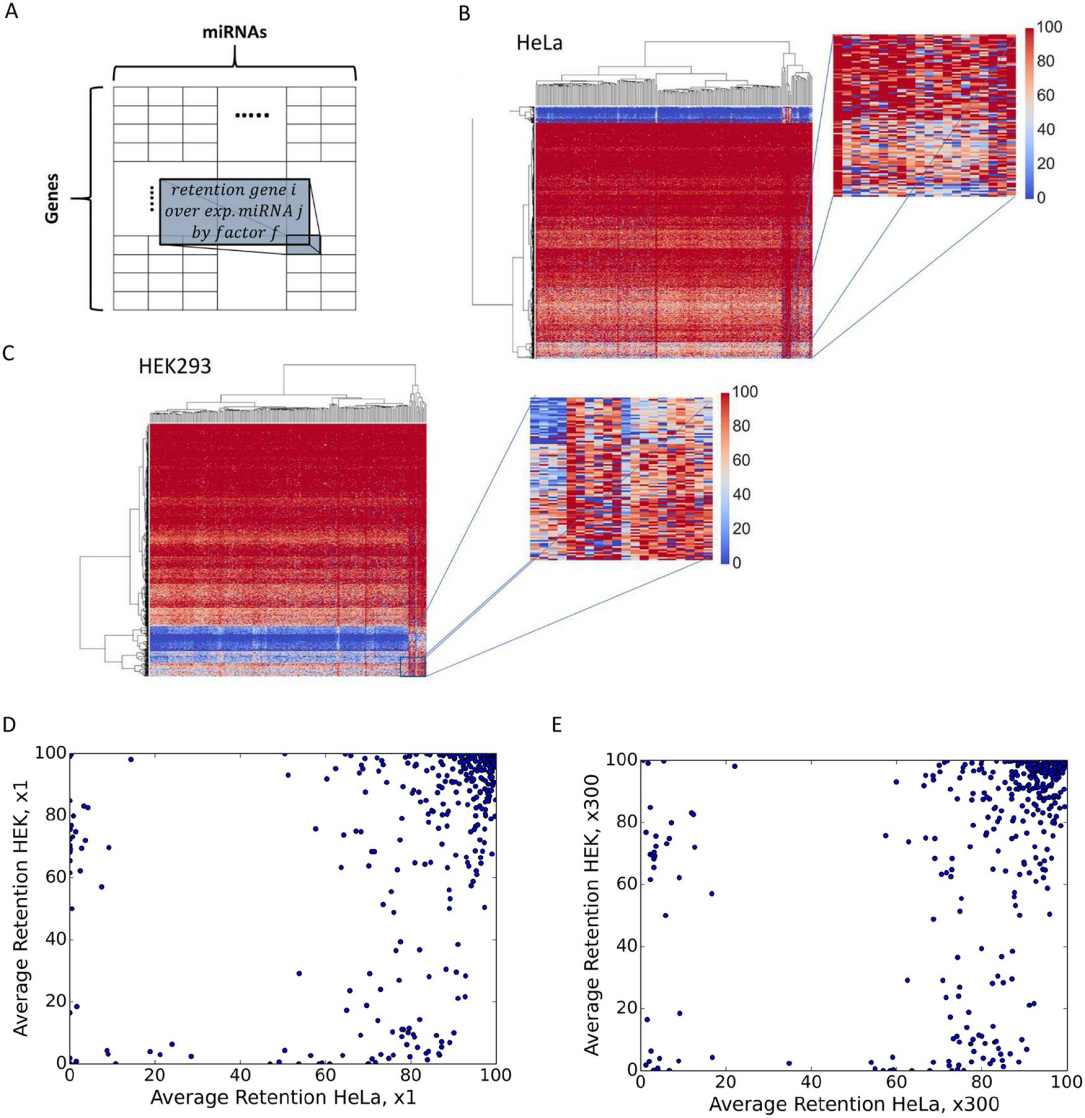
miRNA overexpression matrices. **(A)** The columns stand for the different miRNAs overexpressed by factor f, and the rows stand for the different genes above a predetermined expression level. **(B)** Heatmap of the retention range (in %) for genes where all miRNAs are overexpressed at a factor x300. Each row is associated with a specific gene nad the clustering is performed by the rows (i.e. genes). The matrix includes 248 expressed miRNAs in HeLa cells. **(C)** Zoom-in of a small section of the heatmap of the retention range for genes that were overexpressed at a factor x300 as shown in B. Each row is associated with the retention by overexpressing any of the miRNAs. **(D)** HeLa and HEK-293 average final retention comparison in a control setting (x1). Each point stands for a shared gene in the 248-overexpression conditions (a row in the heatmap presented in **B, C**). **(E)** HeLa and HEK-293 average final retention comparison as in D for the over expression factor x300.

To determine whether the composition and stoichiometry of miRNAs and mRNAs dictate miRNA regulatory behavior, we performed exhaustive and systematic manipulations of all cellular miRNAs (see S5 Dataset for miRNA profiles in different cell lines). We first clustered individual miRNAs according to their families. Altogether there are 248 such families in HeLa cells that match their representation in the miRNA-MBS TargetScan prediction matrix (see Materials and methods). We multiply the basal abundance (x1) of each of miRNA families by a factor (*f*) to get matrices of retention values of genes (rows) and miRNA (columns, 248 families). As each miRNA family was overexpressed by the tested factor (*f*), we obtained a series of matrices for each factor (x3, x9, x18, x90, x300 and x1000). Thus, matrix M*f*_ij_ is the final retention of gene *i* after 100k iterations of COMICS for the overexpressed experiment of miRNA *j* (Fig 5A). For an unexpressed miRNA, a minimum level of expression is assigned as its initial level (x1 level, see Materials and methods). For clarity, the matrix M*f*_ij_ in Fig 5 is restricted to genes whose initial expression level exceed a pre-determined threshold (e.g., 775 rows for HeLa cells, S6 Dataset).

Inspecting M*f*_ij_ for each overexpression condition reveals the presence of a substantial set of genes that are characterized by high retention >85% for ≥90% of the tested miRNAs (i.e., the high retention criterion satisfied by at least 225 of the 248 tested miRNA families). We refer to this set as cross-miRNAs stable genes. The number of stable genes is 185 genes for HeLa, 176 genes for HEK-293 and 123 genes for MCF-7. Running COMICS on HeLa cells for 200k iterations, instead of the default 100k setting, obviously resulted in fewer number of genes with high retention (<85% retention, 71% of the genes when compared to 100k iteration run). However, testing the set of cross miRNA stable set validated that 94% (176 out of 185 in Hela) of the genes overlap, validating the robustness of the gene resulting lists to the parameters of COMICS run. A full detailed analysis of cross-miRNAs stable genes is available in S7 Dataset. These unexpected observations imply that a set of genes in each cell type is resistant to miRNA-dependent attenuation of gene expression, regardless of the actual identity of the miRNA. Such coordinated, concerted action of miRNAs seems to be valid for any tested cell type, and to the best of our knowledge was not described previously.

The matrix M*f* also reveals a small well-defined gene set that is highly sensitive to miRNA regulation. Specifically, these are genes with a retention rate below 50% for ≥90% of the tested miRNAs among all overexpression experiments. These genes are referred to as cross-miRNAs sensitive genes. We report on the sensitive genes for HeLa (23 genes), HEK-293 (34 genes) and MCF-7 (22 genes). For a full detailed analysis of cross-miRNAs sensitive genes see S8 Dataset. These results imply that a small set of genes in each cell type is prone to regulation by (almost) any overexpressed miRNA. Therefore, attenuation in gene expression is expected for a small set of genes regardless of the actual miRNAs’ composition. Changing the definition for stable and sensitive gene list had a negligible effect on the resulting list and the downstream analysis (S1 Text).

For illustration, the matrices [Mfij (x300)] for HeLa (Fig 5B) and HEK-293 (Fig 5C) are colored to indicate genes with high (red) and low retention (blue) levels. The matrices represent clustering by genes and miRNAs, where the clustering dendrogram highlights the emergence of a strong signal for the sensitive genes (blue rows) in both cell types. Still, a remarkable richness in the retention pattern is associated with each gene and miRNA (Fig 5C, zoom in, Mf_ij_ x300). The miRNAs that are naturally clustered by their similar profile across all genes (shown by a coherent pattern across columns) will not be further discussed.

We then tested whether the characteristic of the retention profiles is persists across different cell types. Fig 5D and 5E compare the average retention observed for each of the shared genes from HeLa and HEK-293. A large difference is observed in the distribution of genes for HeLa and HEK-293 when profiles in Mf_ij_ (x1) and Mf_ij_ (x300) are compared. This global view suggests that the same miRNA manipulations drive different cell types to greatly differing end-state.

### Identifying a set of genes that are insensitive to miRNA manipulations in different cells

**Fig 6:**
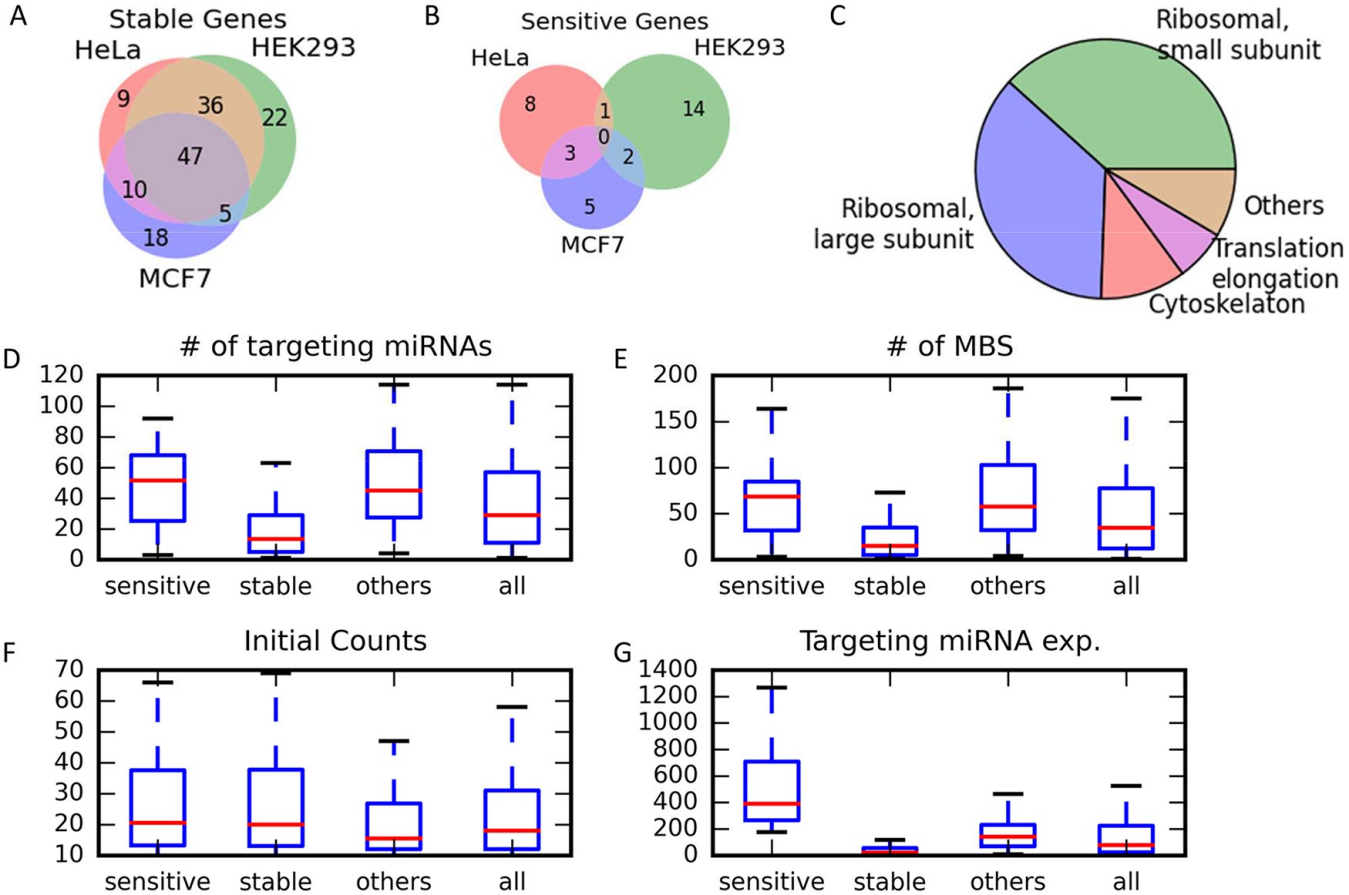
Comparison of sensitive and stable gene sets in different cell types. **(A)** Overlap of the cross-miRNA stable genes in HeLa, HEK-293 and MCF-7 cells. Only genes that are expressed in at least two cells are listed. The gene list of the stable genes is available in S7 Dataset. **(B)** Overlap of the cross-miRNA sensitive genes in HeLa, HEK-293 and MCF-7 cells. Only genes that are expressed in at least two cells are listed. The gene list of the stable genes is available in S8 Dataset. **(C)** The 48 stable genes shared by HeLa, HEK-293 and MCF-7 cells according to their functional annotations: (i) small ribosomal subunit (18 genes), (ii) large ribosomal subunit (17 genes), (iii) cytoskeleton (5 genes), (iv) translation elongation (3 genes) (v) 4 additional genes. For detailed list see S9 Dataset. **(D-G)** Comparison of the number of targeting miRNA of sensitive genes, stable genes, and others (not sensitive and not stable in HeLa cells). **(D)** Statistics of the comparisons are significant for the comparison of stable genes set and both sensitive and other genes (t-test p-values of 7.53e-11 and 6.48e-21, respectively). No significant difference between sensitive and other genes. **(E)** Comparison of the number of MBS of sensitive genes in HeLa cells. Statistics of the comparisons are significant for the comparison of stable genes set and both sensitive and other gene sets (t-test p-values of 2.07e-9 and 5.6e-15, respectively). No significant difference between sensitive and other genes. **(F)** Comparison of the initial abundance of sensitive genes. Statistics of the comparisons are significant for the comparison of stable genes set and both sensitive and other genes (t-test p-values of 0.017 and 0.015, respectively), and no significant difference between sensitive and other genes. **(G)** Comparison of the average expression of the targeting miRNAs of each gene in HeLa cells. Significant differences between all three gene sets were found (t-test p-values 2.52e-22, 3.07e-9 and 8.7e-11 for the comparison of stable-sensitive, stable-others and sensitive-others, respectively). Full statistics are shown in Supplemental S2 Table.

To better characterize the cross-miRNA stable genes, we tested their correspondence in each of the analyzed cell types. Fig 6A shows the unified pattern for Mf_ij_ that was found in HeLa, HEK-293, and MCF-7 for the cross-miRNAs stable and sensitive gene sets. Note that the analysis is limited by the subset of genes common to all three cell types, and to those genes with expression level exceeding a predetermined threshold (>0.04% of expressing mRNA molecules). From 78 (MCF-7), 102 (HeLa) and 110 genes (HEK-293) defined as cross-miRNAs stable genes, 48 genes are common to all three cell types. The overlap among all three gene lists is very significant (Chi-Square test, p-value = 1.35e-08, Fig 6A). It argues that the stable genes are immune to miRNA regulation under a wide range of overexpression settings and across numerous cell types, a phenomenon that corroborates the notion of a concerted action of miRNAs in each cell types. The list of shared 48 genes is shown in S9 Dataset.

A similar analysis that was performed on the cross-miRNAs sensitive gene lists (S8 Dataset) resulted in an opposite trend. Not only is such gene set far smaller (Fig 6B). No shared genes appear as sensitive in these three cell types.

We tested whether the computational discovery of stable and sensitive gene sets is corroborated by our cellular experiments. To this end, we created computational-based and experimental-based lists and tested the statistical significance for the overlap of such lists by applying a hypergeometric test. For COMICS, we considered genes that exhibit a coherent retention level for at least 90% of the overexpressing miRNAs, and meet the threshold of >85% retention for stable, and <50% for sensitive genes. For the experimental-based lists, we collect genes that meet the same % retention threshold for gene expression ratio at 24 hrs relative to 0 hr. At 100k iterations COMICS and HeLa cells stable list significantly overlap (p-value = 0.00064, S5 Fig). For HEK-293, the sensitive list displays maximal significance. However, throughout the COMICS run (50k iteration intervals, 20 hypergeometric tests). the calculated p-values remain significant, ranging from 1e-3 to 1e-10 (S5 Fig). Despite the strong statistical overlap of the COMICS and the experimental results, fundamental differences exist between the computational and the experimental settings. Specifically, numerous processes that regulate mRNA degradation in living cells (e.g., poly-A shortening, decapping) are not explicitly implemented in COMICS. Nevertheless, a strong resemblance is measured between genes lists exposes understudied design principle for miRNA regulation in cells.

### The cross-miRNAs stable set is enriched in genes of the translation machinery

We applied annotation enrichment tools (see Materials and methods) to the set of cross-miRNA stable genes from HeLa cells (185 stable genes, S7 Dataset). We found that these genes are extremely enriched in Gene Ontology (GO terms) associated with numerous aspects of translation, including translational elongation (GO:0006414), mitochondrial translation (GO:0032543), SRP-dependent co-translational protein targeting to membrane, translational termination (GO:0006415) and more. Combining coherent annotations yield statistically strong enrichment signal (DAVID tool, corrected FDR p-value = 1e-77 to 1e-53) for ribosome structure, elongation machinery, and translational fidelity. Notably, enrichment of annotations associated with protein translation and translation machinery applies for the cross-miRNA stable gene lists from all three cell types (S10 Dataset).

We further tested the enrichment of these genes in biological pathways according to the Reactome database. We observed a large collection of pathways that signifies the lists from each cell type (185, 176 and 123 cross-miRNAs stable genes in HeLa, HEK-293 and MCF-7, respectively). Actually, over 20 translation related pathways are shared among the stable genes of each of cell type. Examples for such pathways include GTP hydrolysis and joining of the 60S ribosomal subunit (FDR ranges from e-80 to e-61), Eukaryotic translation elongation (FDR ranges from e-77 to e-65), Cap-dependent translation initiation (FDR ranges from e-70 to e-60) and more. For a comprehensive list of enriched Reactome pathways see S10 Dataset.

Inspecting annotation enrichment for the set of genes that were identified as cross-miRNA sensitive genes reveal no functional coherence when the individual lists from the different cell-types were compared (S10 Dataset).

The correspondence between the computationally derived stable and sensitive gene lists with the cell experiments yields statistically significant results (S5 Fig). We applied an indirect test to determine whether the function of the high retention genes in the experimental setting agrees with functions associated with COMICS results. We performed an enrichment test based on the collection of Reactome pathways. All expressed genes from overexpression hsa-miR-155 in HeLa cells were sorted by their retention percentage. The top 10% (1192 genes of 11924 listed genes, S1 Table) were subjected to annotation enrichment test. Many of the enriched pathways are associated with translation machinery (FDR ranges from 1e-13 to 1e-6). We conclude that functional overlap is evident for stable genes that are reported experimentally and computationally. It provides a further support for the validity of COMICS to reflect trends of miRNA regulation in living cells.

Fig 6C focuses on annotation partition for the 48 genes that are common to all three cell lines (Supplemental S9 Dataset). The dominant role of translational machinery (Annotation clustering enrichment score ~1e-49) unified annotations of translation elongation and cytosolic ribosome (FDR p-value of 1.18e-67 and 9.36e-60, respectively). Translational machinery component with small and large subunits (35 genes), elongation factors (EIF4A1, EEF1D, EEF1B2) and nucleolin (NCL) account for 79% of the genes in the list. Many of these genes play a role in ribosome production and its dynamics as replicated in the Reactome enriched pathways (S10 Dataset).

We conclude that the cross-miRNA stable gene set signifies the translational machinery. Specifically, the translational machinery highlights a functional gene set that is immune to the regulatory layer of miRNAs. This observation applies to all tested cells and proposes an overlooked cellular robustness to miRNA perturbations.

Finally, we tested the properties that characterize genes associated with the cross-miRNA stable and sensitive gene sets with respect to all other genes (S7 and S8 Datasets). Four properties were tested: (i) the number of targeting miRNAs (Fig 6D), (ii) the number of MBS (Fig 6E), (iii) the initial expression level (Fig 6F), and (iv) the binding potential according to the expression of the most dominant miRNAs in each cell type (Fig 6G, S5 Dataset). Recall that features (iii-iv) are cell specific. We observed that genes belonging to the stable set are characterized by smaller number of MBS and smaller number of targeting miRNA relative to other genes (t-test = 6.48e-21 and 5.64e-15, respectively). While the statistic for the initial expression levels of these genes is marginal for all cell types, the most significant differentiating feature between the stable and sensitive gene sets is the targeting potency by the most abundant miRNAs in cells (t-test = 2.52e-22, Fig 6G). For example, The most abundant miRNA expressed in HEK-293 is hsa-mir-7 (25% of total miRNA molecules). While it targets only 3.5% of the stable genes, it can bind to 94% of the cross-miRNA sensitive gene set. Therefore, we conclude that stable genes are inherently resistant to regulation by any of the most abundant miRNAs, despite the presence of MBS to low expressing miRNAs. The S2 Table lists the detailed t-test statistics for the feature-based analysis of all three cell lines.

We claim that the exhaustive and unbiased comparison between the stable and sensitive genes reveals an overlooked signal referring to the nature of the MBS in view of their gene functions. Despite the great difference in overall miRNA composition of different cell lines, several miRNAs are shared across many cell-types (e.g., hsa-mir-21, hsa-let-7 and hsa-mir-92). In all tested cells, the MBS for these genes are excluded from the genes of the translational machinery. Therefore, various cellular systems are intrinsically immune to fluctuations in translation by abundant miRNAs.

## Discussion

### miRNAs stability as a major determinant in cell regulation

Direct measurements of miRNA and mRNA composition in cells cannot trivially predict their behavior [13, 39]. Detailed quantitative consideration of miRNA and mRNA governs the dynamics and the steady state of the expressed genes [21, 40]. Nevertheless, the underlying rules for post-transcriptional regulation by miRNAs are still missing [41].

We studied cells’ response to miRNA regulation under a simplified condition of transcriptional arrest by testing the retention profiles of mRNAs as readout. Upon such condition, the dominant effect of miRNAs is most likely via attenuation of mRNA stability rather than by translation repression [42]. As most miRNAs are transcribed by RNA PolII from their own promoters, it is essential to account for the effect of transcription arrest on miRNA abundance. The results in Fig 1B, S1 and S2 Figs show that miRNAs are extremely stable at least during the 24 hrs after the cells were exposed to ActD. In all studied systems, stabilization of miRNAs is attributed to the protecting capacity of AGO-2 [43]. The number of AGO-2 molecules in cells is insensitive to transcriptional arrest [44]. Our results further show that despite the presence of ActD, AGO-2 is not limiting in the system (Fig 1).

The starting point for COMICS simulation is the gene expression pattern as measured experimentally in different cell types (Fig 1, S1 and S2 Figs). COMICS simulation considers a molecular ratio of 2:1 between miRNAs and mRNAs, under the assumption of excess in AGO-2. The probabilistic analysis of cells under varying levels of miRNA overexpression argues that AGO-2 occupancy is not a limiting factor. Actually, a change in the number of molecules, while maintaining the stoichiometry impacts mostly on the dynamic of miRNA-mRNA pairing (S1 Text, S6 Fig).

### COMICS is robust towards a broad range of parameters

We tested the reliability of COMICS to accurately reflect the miRNA-mRNA competition in living cells. To this end, we altered extensively the simulator’s operational parameters and assessed the sensitivity and robustness of the results (File S1 Text). Overall, we demonstrated the robustness of COMICS by varying a large number of parameters (e.g., number of COMICS iterations, S4, S5 and S6A Figs; Stoichiometry of miRNAs and mRNAs, S6B and S6C Fig; Simulator intervals for mRNA degradation, S6D Fig; Interaction scoring tables, S7 Fig). Recall that in living cells additional processes take place for dictating the half-life and stability of cytoplasmic mRNAs. These processes involve the activity of several exoribonucleases, decapping enzyme, RNA modifying enzymes, as well as RNA secondary structure and localization [45]. Evidently, such miRNA-independent processes are not explicitly modelled by COMICS. Moreover, we validated the dependency of COMICS outcome on the quality of the miRNA-MBS table of interactions. Moreover, the sampling protocol of miRNAs and mRNAs was altered without effecting the overall outcome (S7 Fig). The used interaction table integrates a rich body of knowledge on miRNA specificity and affinity according to computational and experimental evidence [27].

There are several natural extensions to COMICS that we intend to explore in the future. This includes the formulation of a synergistic cooperativity of miRNA binding at non-overlapping MBS [16, 46], allowing options for rationally induce preselected mRNAs, and including parameters associated with the ceRNA paradigm. In general, COMICS framework is attractive for testing unresolved questions and emerging principles of miRNA regulation in vivo [21, 32, 37].

### miRNA composition is a major determinant in establishing cell identity

We developed COMICS platform to handle scenarios where cells undergo drastic changes in their miRNA composition. Altogether, the results of thousands of simulation processes were completed to test the impact of alteration in the expression of hundreds miRNA families (Fig 5). We were able to describe general trends from these simulations that apply to three different cell types (S8 Fig). Importantly, each of the tested cell expresses a different profile of miRNAs, supporting the notion of a unique miRNA profile determines the cell identity [47] (S1 Text, S8B Fig). Indeed, a transition between cell types is attributed to the presence and expression level of a specific miRNA (e.g. miR-34a [48]). Similarly, the actual miRNA profiles are associated with the establishment of cell malignant states [49, 50].

Two extreme miRNA regulation trends were revealed in this study. Firstly, the robustness of a translation apparatus to (almost) any miRNAs. Secondly, the extreme sensitivity of a cell-specific small set of genes to (almost) any miRNAs. The identification of a small set of genes (about 3% of the reported genes) that is exceptionally sensitive to miRNA regulation, is surprising as it predicts that down-regulation of gene expression will result from altering the expression of almost any miRNA (Fig 6B). Remarkably, many of the sensitive genes are nuclear, and play a role in transcription regulation, nuclear function and a collection of cell processes (S10 Dataset). Over-enrichment in MBS for the most abundant miRNAs signify many of these sensitive genes (S2 Table). For example, targeting by hsa-mir-21 is prevalent among the cross-miRNA sensitive genes. The hsa-mir-21 occupies 27% and 32% of total miRNAs in naive MCF-7 and HeLa cells, respectively (S5 Dataset, S8A Fig). The hsa-mir-21 occupies 58 and 73 MBS among the sensitive genes identified in MCF-7 (list of 22 genes) and HEK-293 (list of 34 genes), respectively.

We plan to extend the use of COMICS to a large collection of human cell lines (e.g., NCI-60) for assessing the generality of our findings. Specifically, applying COMICS simulations to cells that are signified by metastatic potential is attractive for planning specific miRNA manipulation for a desired outcome.

### Characteristics of genes with respect to the most abundant miRNAs

A relatively large gene set (about 20% of the reported genes) that is exceptionally stable to almost any miRNA manipulation, irrespectively to the actual levels of the miRNA in cells is unexpected (S6 and S8 Datasets). Based on the statistically significant functional overlap among three cell types it is postulated that the stable genes share unified feature in their 3’-UTR sequences. Indeed, the most significant difference between the features associated with the sensitive and stable sets (p-value = 1.57e-23) concerns the cellular level of miRNA expression levels (Fig 6G). In cancer cells, the most abundant miRNA genes (e.g., hsa-mir-21, hsa-mir-30, hsa-mir-15/16) are involved in cancer development. In these instances, a minor change in the expression of such miRNAs leads to a cell state transition [51]. In view of our findings, we propose that at least in humans, the ribosomal proteins and various components of the translation machinery became robust to miRNA regulation. Eventually, many of the ribosomal subunits were not included in the stable gene list with some ribosomal proteins show sensitivity to miRNA regulation, other ribosomal proteins may have very short 3’-UTR and no MBS. The later genes are completely excluded in our analysis. Throughout the analysis we only considered genes that are reported with high confidence MBS by the TargetScan probability matrix (see Materials and methods). We propose that genes of the translation machinery achieve a maximal robustness vis-a-vis miRNA regulation. The outcome is that genes that play role in translation are practically resistant to changes even in the presence of high expressing miRNAs.

In summary, the immunity of the translation system to miRNA regulation suggests that it may be part of a global cell strategy [52]. Evidently, there is a fundamental difference between transcription and translation processes. While the transcription system can quickly respond to the needs dictated by abrupt changes in the environment, the translational machinery is very costly and slow responding, and as such is less prone to variations. Therefore, sustaining an immunity towards the majority of miRNAs, including the high expressing ones unveil an overlooked design principle in miRNA regulation. The driving force of evolution, acting on targets and their MBS underlying the properties of the miRNA network in many organisms [53]. It is for the future to investigate whether this evolutionary refinement of 3’-UTR of the components of the translation apparatus can be generalized to other organisms along the phylogenetic tree.

## Materials and methods

### Cell culture

Human cell line of HeLa (cervix epithelial, # CCL-2) and HEK-293 (embryonic kidney, # CRL-1573) were purchased from the cell-line collection of ATCC. Cells were cultured at 37°C, 5% CO_2_ in Dulbecco's Modified Eagle Media (DMEM, Sigma), supplemented with 10% FBS (Life Technologies), and 1% antibiotics mixture (Sigma-Aldrich, Cat # P4333). Cells were maintained for 2 weeks and passing and splitting cells was carried out at 70–80% confluence.

### Transcription arrest and miRNA overexpression

Overexpression of miRNAs was performed by transfected HeLa cells and HEK-293 with miRNA expression vectors that are based on the miR-Vec system, under the control of CMV promotor (Origene). Cell transfection was done using Lipofectamine 3000 (Invitrogen) as described by the manufacturer. Cells at 70% to 80% confluency were transfected with 1.5μg purified plasmid DNA containing hsa-mir-155 and hsa-mir-124a (kindly contributed by Noam Shomron, Tel Aviv University). Control empty vector expressing GFP (0.15μg) was mixed with the CMV-miR expressing vectors. Cells were monitored by fluorescent microscopy at 36 and 48 hrs post transfection. The efficiency of cell transfection was >75% of the HeLa cells and ~100% of the HEK-293 according to the GFP expression at 48 hrs post transfection. Transcription inhibition was achieved by adding to cultured HeLa and HEK-293 cells media containing Actinomycin D (ActD, 10 μg/ml in DMSO), or the appropriate control (i.e. DMSO). Cells were treated with ActD (10 μg/mL, Sigma) 24 hrs post-transfection. Cells were cultured in 6-well plates and following treatment were lysed in 1 ml TRIzol (Invitrogen) at the indicated time points (0 hrs, 2 hrs, 8 hrs, 24 hrs).

### Library preparations for deep sequencing

Purification of total RNA containing miRNA extracted from ~2*10^6^ cells using QIAzol Lysis Reagent RNeasy plus Universal Mini Kit (QIAGEN, GmbH, Hilden, Germany). To ensure homogenization a QIAshredder (QIAGEN, GmbH, Hilden, Germany) mini-spin column has been used. Sample has been transferred up to RNeasy Mini spin column and centrifuge for 15s at ≥8000g at room temperature, and the mixture was processed according to the manufacturer’s standard protocol. Samples with an RNA Integrity Number (RIN) >8.5, as measured by Agilent 2100 Bioanalyzer, were considered for further analysis. mRNA libraries were generated using the Illumina Truseq RNA V2 library Seq protocols.

For small RNA library construction, ~1 μg of RNA was used. RNA was ethanol precipitated to enrich for small RNA. Small RNA libraries were prepared according to NEBNext Small RNA Library Prep Set for Illumina (Multiplex Compatible) Library Preparation Manual. Adaptors were then ligated to the 5’ and 3’ ends of the RNA, and cDNA was prepared from the ligated RNA and amplified to prepare the sequencing library. The amplified sequences were purified on 4% E-Gel Agarose gels (ThermoFisher # G401004), and sequences representing RNA <200 nt were extracted. Data used are derived from at least two biological duplicates. The average values of the two independent sets are reported.

### RNA deep sequencing analysis

RNA extracted from HeLa and HEK293 cells were taken from independent library preparations and were processed in the same sequencing slides according to standard Illumina Protocol [54]. Deep sequencing was performed on small RNA (<200 nt) molecules and for mRNA by standard RNA-Seq Illumina Protocol. Each of the 48 RNA-sequencing samples covers the mRNA and miRNA sets (24 sets for the ActD treated on two cells types, at 4 times points with two sets of miRNA overexpression and a set for the control transfected by an empty vector). Each sample consisting of ~25M total reads of length 100 for each read for mRNA detection, and ~10M total reads for the miRNA detection. The sequencing data was processed by removal the adaptors and filtering out low quality sequences. The filtered high-quality fragments were mapped to the human transcriptome of hg19 gtf file from UCSC provided by Galaxy. Specifically, the sequenced small RNAs were trimmed using Cutadapt ver. 1.13 and quality filtered using FASTX toolkit. Short reads (~30 nt long) were mapped to miRNA using mapped to miRNA genes using miRExpress 2.0 [55]. Longer reads were aligned against human genome hg19 using TopHat 2.1.1 under default flags. For mRNA expression evaluation, mapped reads were submitted to Cufflinks toolkit version 2.2.1. Out of the mapped reads, only reads of length > = 17 were considered. miRNA sequences refer to mapped, high quality reads that are aligned to any of the pre-miRNA as defined by miRbase databases (ver. 21.0) [56].

### Normalizations of mRNA expression and miRNA families

For analysis of all experimentally tested samples an estimation of mRNA molecules per cell was assigned to 25,000 molecules at time 0 (25k, prior to activation of the transcription inhibition protocol). Ten of the highly expressed genes were selected from the top ranked list of mRNAs. These genes were selected as being stable throughout the 24 hrs of the ActD protocol, along all four time points and were considered anchor genes. According to their quantification and the total quantity of the gene expression distribution a correction was implemented based on these anchored genes. The listed values for mRNAs (S1 and S2 Datasets) are based on pair-end sequencing protocol and calculated as FPKM. miRNAs are counted as TPM (S5 Dataset). For the rest of the analysis, reported genes are those with an overall expression which is above the threshold of 5 molecules after the quantification correction procedure (>0.02% expression). For miRNA normalization we estimated 50,000 molecules per cells and only miRNAs with more than 1 molecule after quantification were considered. The identified miRNAs were compiled to their families. This transformation was applied to the TargetScan scoring tables and the most significant score of miRNA representative was assigned to its family.

### miRNA in silico manipulations

Overexpression scheme is based on multiplication of the available miRNA amount by numerous factors (from x1 to x1000). The addition of miRNA molecules calls for re-calculating a new miRNA distribution while fixing the amount of miRNA in the cell. In case that a specific miRNA had not been detected in the native cell, an arbitrary starting minimal amount of 0.01% (the equivalent of 5 molecule/ cell) is considered.

### TargetScan probabilistic pairing of miRNA-mRNA

The probabilistic framework interaction table was adapted from the scores provided by TargetScan [27]. Accordingly, high probability of successful interactions is calculated from a combination of strongly supported miRNA-mRNA pairs that comply with features from sequence, secondary structure and evolution conservation. The complete miRNA-mRNA table include 8.22 M pairs that covers also poorly conserved interactions. We compiled the version of TargetScanHuman (Release 7.1) that reports on 19,475 genes (28,353 transcripts). We extracted the TargetScan mRNA CWCS scores (cumulative weighted context++ score), which is a proxy for the predicted repression based on the different properties of the MBS sites. The CWCW estimates the score by compiling the contribution of multiple MBS according to a miRNA family and the relative positioning at the 3’-UTR of the transcript. The predicted repression scores range from 0–1, and are identical for all representation of the relevant miRNA family members [27]. We used a compressed version of the table that report only on pairs that are supported by conserved miRNAs with 1,183,166 pairs, covering 18,953 genes and 289 miRNA families.

### COMICS sampling and iterations

In each run, a random miRNA is chosen from the predetermined available miRNAs distribution. Next, a target is chosen randomly according to the available target distribution. mRNA that is already bounded by other miRNA molecules can be a putative target for the chosen miRNA, if the relevant binding site is not overlapping an occupied MBS on the same molecule. Overlapping binding sites are considered for neighboring MBS that are <50 nucleotides apart. Note that MBS that physically overlap in their sequence are already removed by TargetScan with the notion that overlapping sites cannot be occupied at the same time. A binding event will occur according to the miRNA-mRNA binding probability as extracted from TargetScan interaction table (or other prediction tables). The conversion of the interaction scores to the binding probabilities was done according to TargetScan score: p = 1–2^score^. Upon a binding event, the free miRNA and mRNA distributions are updated, and the bounded mRNA molecules are marked as being occupied. An occupied molecule is removed after 1000 iterations following a successful binding event (a tunable parameter for halting an instant mRNA degradation, S5D Fig). For mRNA to be eliminated, at least one MBS must be reported as occupied. During those iterations, it is eligible to bind other miRNAs in any of its non-overlapping binding sites. After mRNA removal, the bounded miRNAs are released and return back to the free miRNA pool and are suitable for engaging in further binding events.

### Statistics and bioinformatics

P-values were calculated using a paired and unpaired t-test, Fisher exact test, Kolmogorov Smirnov (KS) test, Wilcoxon signed-rank or Chi-square tests. For testing the correspondence of two sets of different sizes, we have used the KS test. Statistical values are based on correlations were performed using standard Python statistical package. Annotation enrichment statistics [57] was used using the Gene Ontology (GO) annotation platform that address Panther protein groups and Reactome pathways. For testing the effect of different background gene lists for an enrichment statistic we applied DAVID [58]. The clustering enrichment score is based on one tail Fisher exact corrected for the number of gene ontology annotations that are used. Enrichment was performed in view of genes that are potential candidates for our analysis and against the set of genes that express with a minimum of 0.02% of the mRNA overall expression. Corrections for multiple hypothesis were applied and FDR results are reported.

## Supporting information

S1 Table Annotation enrichment for the top 10% (1192 genes) from HeLa cells overexpressed with hsa-mir-155, sorted by high retention level (mRNA expression at 24 hrs relative to 0 hr). (XLSX)

S2 Table Summarizes the statistic characteristics of the sensitive and stable sets for 3 cell lines: HEK-293, HeLa and MCF-7. (XLSX)

S1 Fig The expression profile of miRNA and mRNA in HeLa cells under transcription arrest by ActD. **(A)** Expression of miRNAs in pairs of 4 different time points. RNA samples were collected at 0 hr, 2 hrs, 8 hrs and 24 hrs following transcription inhibition by ActD. The scale for the expression levels is in log10 scale. Spearman correlation (r) is listed along the p-value of the significance. **(B)** Expression of mRNAs in pairs of 4 different time points. RNA samples were collected at 0 hr, 2 hrs, 8 hrs and 24 hrs following transcription inhibition by ActD. The scale for the expression levels is in log10 scale. Spearman correlation (r) is listed along the p-value of the significance.
(TIF)

S2 Fig The expression profile of miRNA and mRNA in HEK-293 cells under transcription arrest by ActD. **(A)** Expression of miRNAs in pairs of 4 different time points. RNA samples were collected at 0 hr, 2 hrs, 8 hrs and 24 hrs following transcription inhibition by ActD. The scale for the expression levels is in log10 scale. Spearman correlation (r) is listed along the p-value of the significance. **(B)** Expression of mRNAs in pairs of 4 different time points. RNA samples were collected at 0 hr, 2 hrs, 8 hrs and 24 hrs following transcription inhibition by ActD. The scale for the expression levels is in log10 scale. Spearman correlation (r) is listed along the p-value of the significance.
(TIF)

S3 Fig Retention profile of mRNAs following miRNA overexpressing in HeLa cells. **(A)** Percentage of the genes according to their labels as targets (upper panel, pink) and non-targets (lower panel, blue) according to their retention measured at 24 hrs. **(B)** The plots compare the partition of genes from the control (smooth line), and from hsa-mir-124a overexpressed condition (dashed line). The number of genes that are included in the analyses are marked in parentheses. Target genes are shown in pink lines (top) and the non-target genes are shown in blue lines (bottom). Note the shift in the distribution in the non-target genes towards the genes with higher retention level. All genes with a retention level ≥100 are shown as 100% retention.
(TIF)

S4 Fig The statistical significance of experimental data and COMICS across simulation runs. **(A)** Spearman rank correlation of experimental data for HeLa and HEK-293 following 24 hrs application of ActD. At the beginning of the simulations and following 100k and 1M runs. **(B)** Results from the Wilcoxon signed-rank by -log10(p-value) for the differences in the simulation runs as indicated in the x-axis. The most dynamic section of the difference occurs at the initial 100k iterations. The higher the values, the most significant are the overlap of the gene lists from the experimental and computational settings.
(TIF)

S5 Fig The statistical significance of gene lists derived from experimental data and COMICS. **(A)** The outcome for the cross miRNA-stable and cross-miRNA sensitive sets (marked as stable and sensitive). The COMICS performance is compared in view of the results from the transcription arrest experiment in HeLa **(A)** and HEK-293 cells **(B)**. At each of the indicated steps of the COMICS simulation run, the statistical overlap in gene retention for genes that share their characteristics for >90% of all overexpressed miRNAs. Moreover, stable (defined as >85% retention) or sensitive genes (<50% retention) are calculated. The statistical significance is measured by hypergeometric test with exact p-value which is transformed to -log10(p-value) (y-axis). The higher the values, the most significant are the overlap of the gene lists from the experimental and computational settings. The statistical significance associated with the correspondence of the results are shown at a resolution of each 50k iterations for 1M iteration run (x-axis).
(TIF)

S6 Fig Comparison of different parameter settings of COMICS simulator. **(A)** Pearson correlation coefficients of the final retention after different simulation runs. Each run was conducted using a different set of parameters: different quantification and stoichiometry of miRNA mRNA ratio; different iteration interval between mRNA binding events, and varying the parameter for removal of a mRNA from the system. **(B)** The retention distribution at the end of different runs. The quantity of total mRNAs was fixed to 25k molecules, while its ratio with miRNAs quantity was varying to 1:1, 1:2, 1:4 and 1:8. **(C)** The retention distribution at the end of different runs. The quantity of total miRNAs was fixed to 50,000 molecules, while its ratio with mRNA quantity was set to 2:1, 1:1, 1:2 and 1:4. **(D)** The retention distribution at the end of different runs. The removal interval varied to 1000, 5000 and 10,000 iterations.
(TIF)

S7 Fig Comparison of different simulator runs using different scoring tables. Pearson correlation of different simulation runs using three miRNA-mRNA interaction scoring tables: (i) TargetScan (marked as TS), (ii) randomized table of TargetScan. The randomization was done by fixing the total scores of each gene (row) and each miRNA (column) in the original TargetScan table. (iii) Naïve random table. In this case the original scores of TargetScan table were assigned to random pairing of miRNA–mRNA. Three independent simulation repetitions were performed using each of the above tables. Results for the Pearson rank correlations are color coded. Dark blue indicate correlation of 1.
(TIF)

S8 Fig Abundant miRNA and shuffling cell-specific miRNA profiles. **(A)** Heatmap of miRNA from HeLa, HEK-293 and MCF-7 cell-lines in view of the abundance of each miRNA (colored in log10 scale) in each cell-type. The joint list of miRNAs includes the most abundant miRNAs that occupies 90% of the miRNA molecules (i.e. 45k out of 50k) in each cell-type. The fraction occupied by each of the listed miRNA, for each of the cell-type is available in S5 Dataset. **(B)** Pairs of miRNA and mRNA are shown according to their origin. Pearson correlation of the endpoint of the genes following 100k iterations and testing any of the genes that are above the minimal expression (5 molecules, 0.02% of total amount of mRNA). The number of mRNAs that are considered in the analyses are 516 for the pair of HeLa and HEK-293; 285 for the pair of HeLa and MCF-7 and 305 to the pair of HEK-293 and MCF-7. The p-value of the correlations are very significant for all pairs. All p-values correlation values are < 1e-15.
(TIF)

S1 Text The text covers the design architecture of COMICS as a probabilistic based miRNA-mRNA simulator. It reports on the sensitivity and robustness with respect to numerous parameters’ changes.
(DOCX)

S1 Dataset The mRNAs and miRNAs expression profiles from HeLa cells, and following overexpression of hsa-mir-155. (XLSX)

S2 Dataset The mapped mRNAs and miRNAs expression profiles for the experiment performed in HEK-293 cells. (XLSX)

S3 Dataset The output of the % retention along COMICS 1M iterations at a 10k resolution from HeLa cells with normalized quantities of miRNAs and mRNAs used as input for COMICS iterations. (XLSX)

S4 Dataset COMICS 100k run at a 1k iteration resolution. The output of the % retention is shown for HeLa and HEK-293 cells.
(XLSX)

S5 Dataset Lists of miRNA expression levels for 3 cell types. Used as input for COMICS iterations.
(XLSX)

S6 Dataset The matrix of miRNA retention levels for 248 miRNAs and 775 genes. Source data for Fig 5.
(XLSX)

S7 Dataset Results of the three cross-miRNA stable genes from HeLa, HEK-293 and MCF-7 cells. Only genes that with >5 normalized expression level are listed. The dataset includes a detailed information on the miRNA identity, summary statistics on MBS number and miRNA composition for each gene.
(XLSX)

S8 Dataset Results of the three cross-miRNA sensitive genes genes from HeLa, HEK-293 and MCF-7 cells. Only genes that with >5 normalized expression level are listed. The dataset includes a detailed information on the miRNA identity, summary statistics on MBS number and miRNA composition for each gene.
(XLSX)

S9 Dataset Lists of the unified stable genes shared by HEK-293, HeLa and MCF-7 (48 genes). (XLSX)

S10 Dataset The results from enrichment tests for functional annotation for stable genes that are shared among 3 cell lines (HEK-293, HeLa and MCF-7, 48 genes) and individual stable lists from each of these cell types. Gene annotation list for the sensitive list is presented (based on EnrichR system). The annotation enrichments are based on DAVID scoring method and the Reactome pathway enrichment.
(XLSX)
