# Supplementary figures and images for: A cell-based probabilistic approach unveils the concerted action of miRNAs
Source: PLoS Comput Biol. 2019 Dec 2;15(12):e1007204. doi: 10.1371/journal.pcbi.1007204 (PMC6922470; doi:10.1371/journal.pcbi.1007204)

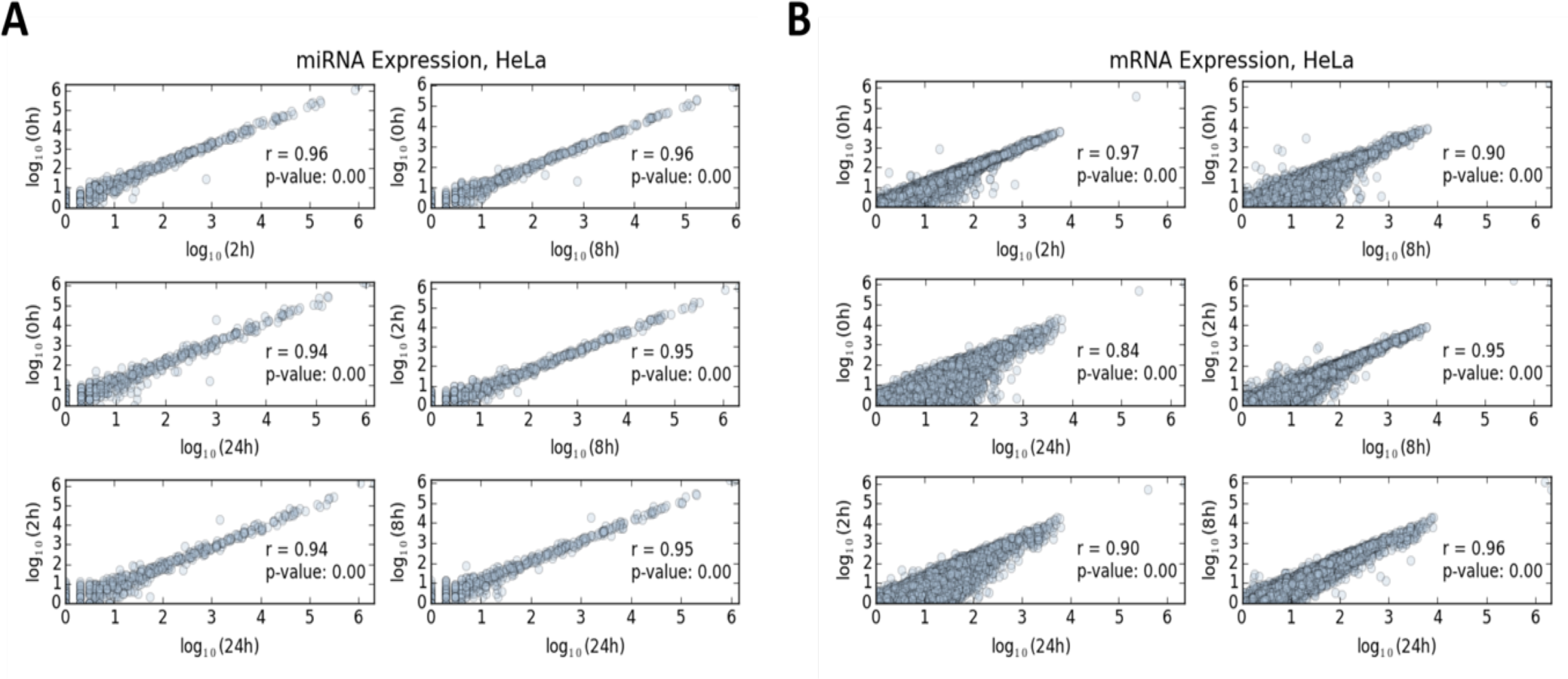

Supplement: S1 Fig — (A) Expression of miRNAs in pairs of 4 different time points. RNA samples were collected at 0 hr, 2 hrs, 8 hrs and 24 hrs following transcription inhibition by ActD. The scale for the expression levels is in log10 scale. Spearman correlation (r) is listed along the p-value of the significance. (B) Expression of mRNAs in pairs of 4 different time points. RNA samples were collected at 0 hr, 2 hrs, 8 hrs and 24 hrs following transcription inhibition by ActD. The scale for the expression levels is in log10 scale. Spearman correlation (r) is listed along the p-value of the significance. (TIF) [file pcbi.1007204.s003.tif]

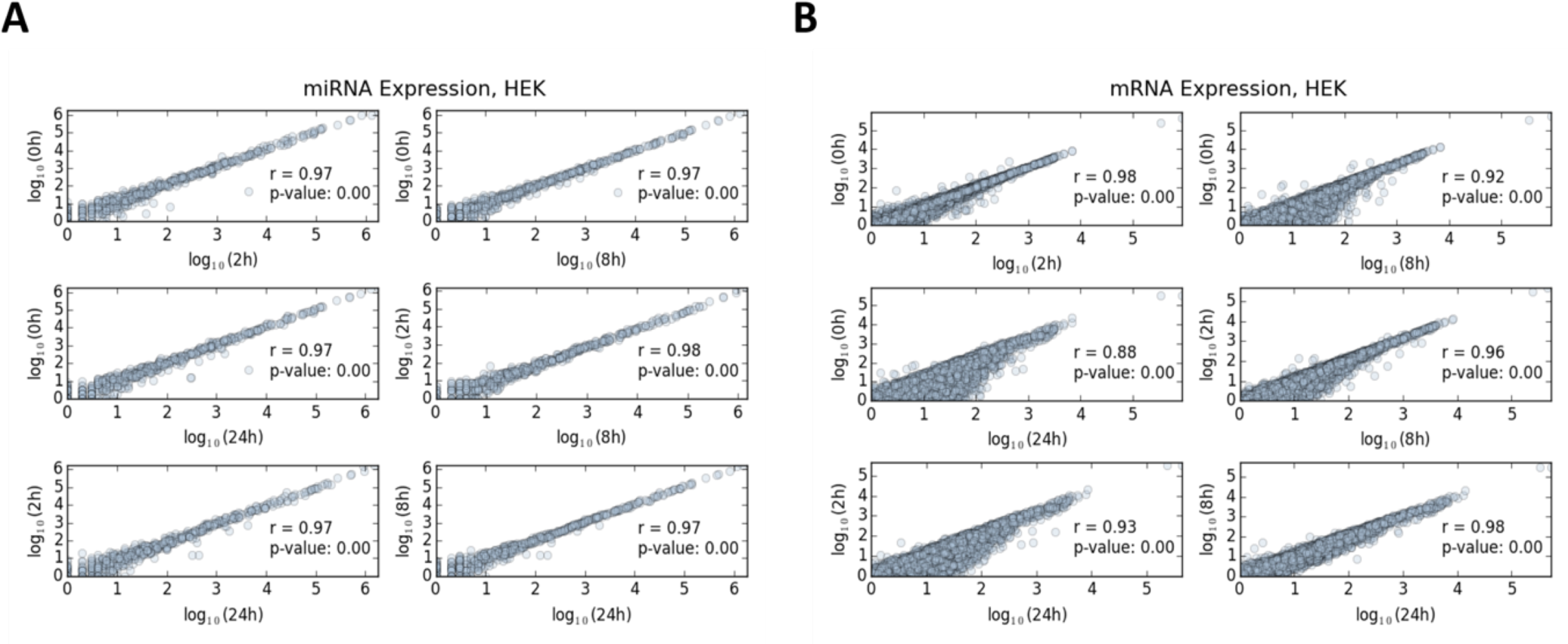

Supplement: S2 Fig — (A) Expression of miRNAs in pairs of 4 different time points. RNA samples were collected at 0 hr, 2 hrs, 8 hrs and 24 hrs following transcription inhibition by ActD. The scale for the expression levels is in log10 scale. Spearman correlation (r) is listed along the p-value of the significance. (B) Expression of mRNAs in pairs of 4 different time points. RNA samples were collected at 0 hr, 2 hrs, 8 hrs and 24 hrs following transcription inhibition by ActD. The scale for the expression levels is in log10 scale. Spearman correlation (r) is listed along the p-value of the significance. (TIF) [file pcbi.1007204.s004.tif]

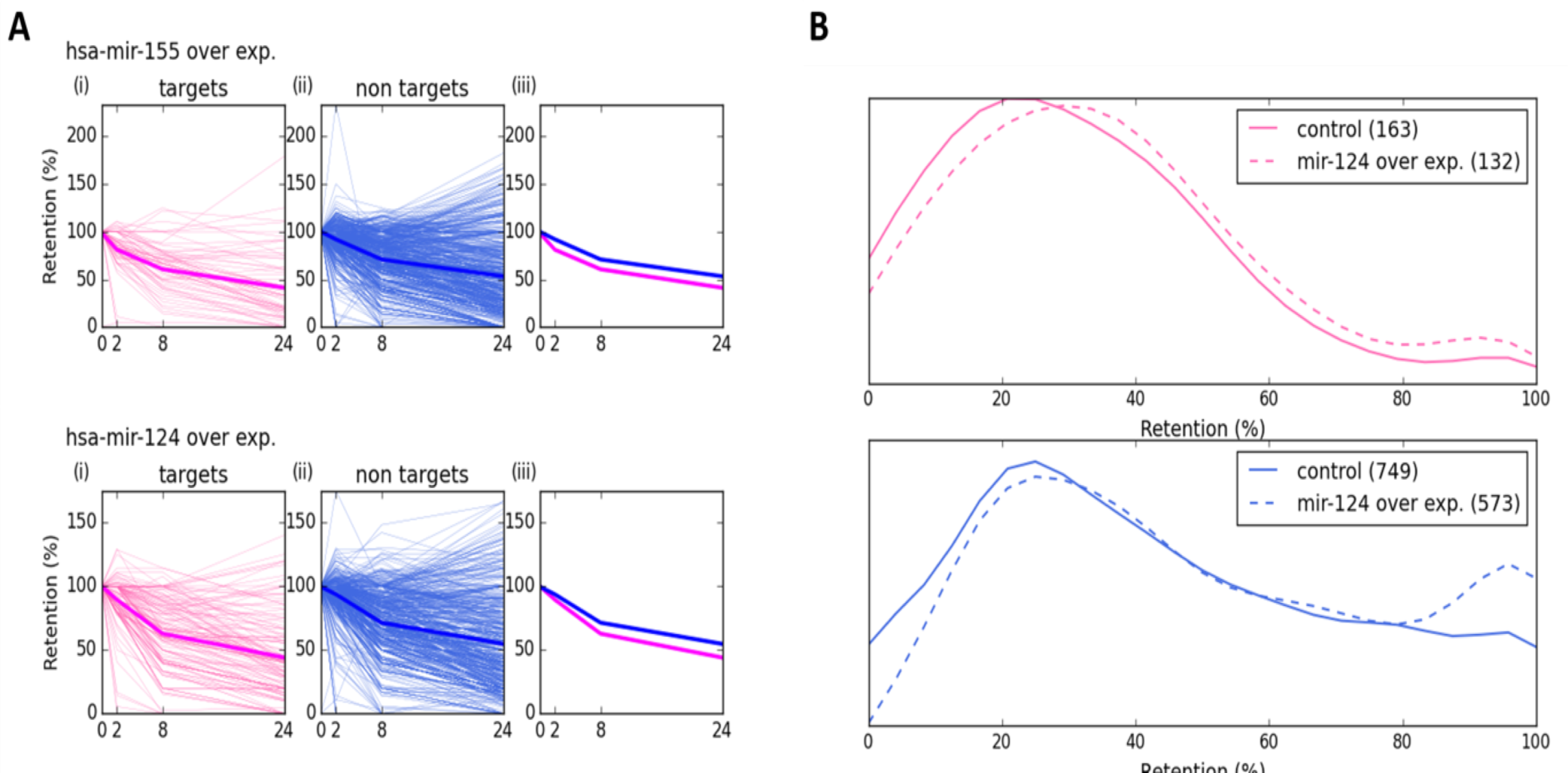

Supplement: S3 Fig — (A) Percentage of the genes according to their labels as targets (upper panel, pink) and non-targets (lower panel, blue) according to their retention measured at 24 hrs. (B) The plots compare the partition of genes from the control (smooth line), and from hsa-mir-124a overexpressed condition (dashed line). The number of genes that are included in the analyses are marked in parentheses. Target genes are shown in pink lines (top) and the non-target genes are shown in blue lines (bottom). Note the shift in the distribution in the non-target genes towards the genes with higher retention level. All genes with a retention level ≥100 are shown as 100% retention. (TIF) [file pcbi.1007204.s005.tif]

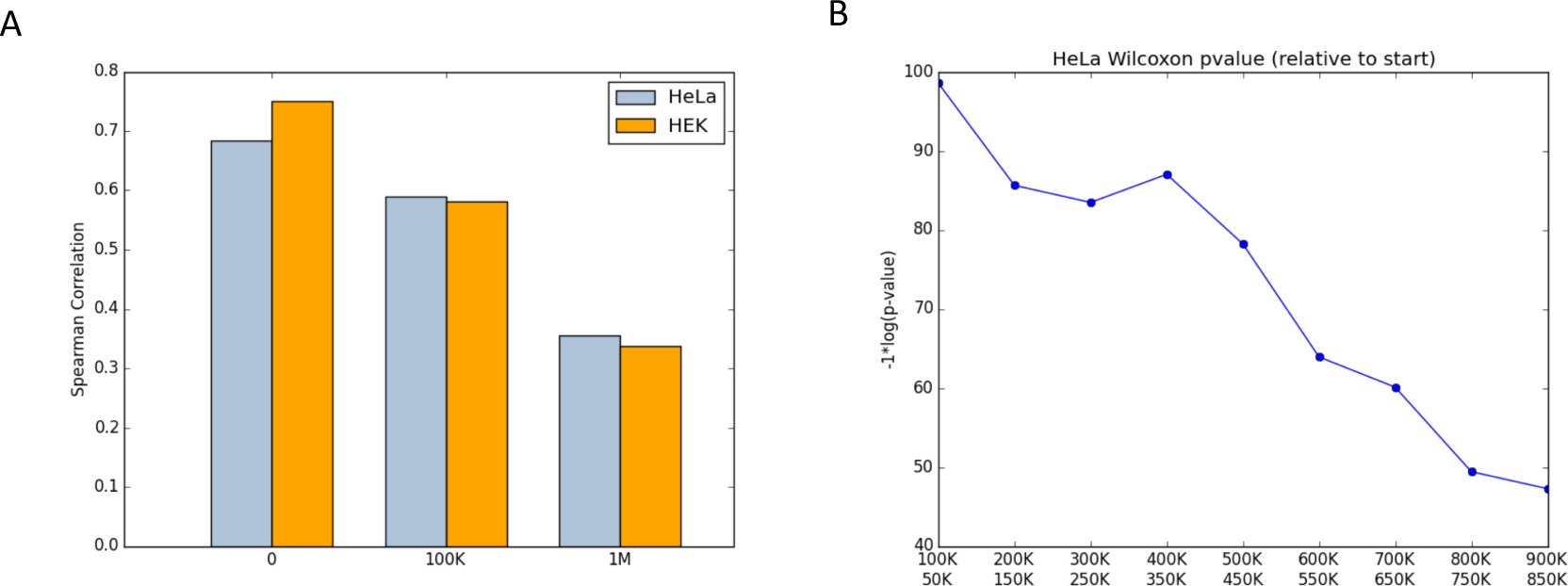

Supplement: S4 Fig — (A) Spearman rank correlation of experimental data for HeLa and HEK-293 following 24 hrs application of ActD. At the beginning of the simulations and following 100k and 1M runs. (B) Results from the Wilcoxon signed-rank by -log10(p-value) for the differences in the simulation runs as indicated in the x-axis. The most dynamic section of the difference occurs at the initial 100k iterations. The higher the values, the most significant are the overlap of the gene lists from the experimental and computational settings. (TIF) [file pcbi.1007204.s006.tif]

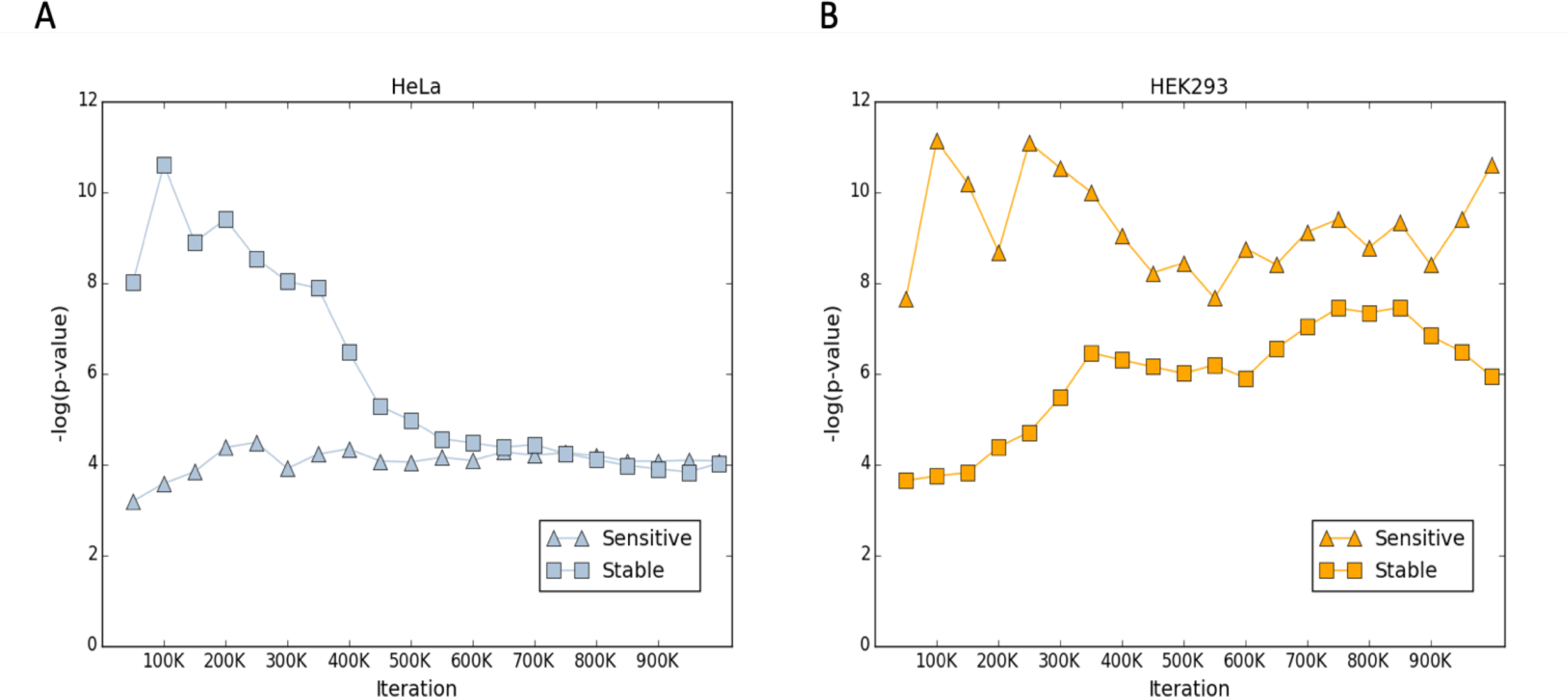

Supplement: S5 Fig — (A) The outcome for the cross miRNA-stable and cross-miRNA sensitive sets (marked as stable and sensitive). The COMICS performance is compared in view of the results from the transcription arrest experiment in HeLa (A) and HEK-293 cells (B). At each of the indicated steps of the COMICS simulation run, the statistical overlap in gene retention for genes that share their characteristics for >90% of all overexpressed miRNAs. Moreover, stable (defined as >85% retention) or sensitive genes (<50% retention) are calculated. The statistical significance is measured by hypergeometric test with exact p-value which is transformed to -log10(p-value) (y-axis). The higher the values, the most significant are the overlap of the gene lists from the experimental and computational settings. The statistical significance associated with the correspondence of the results are shown at a resolution of each 50k iterations for 1M iteration run (x-axis). (TIF) [file pcbi.1007204.s007.tif]

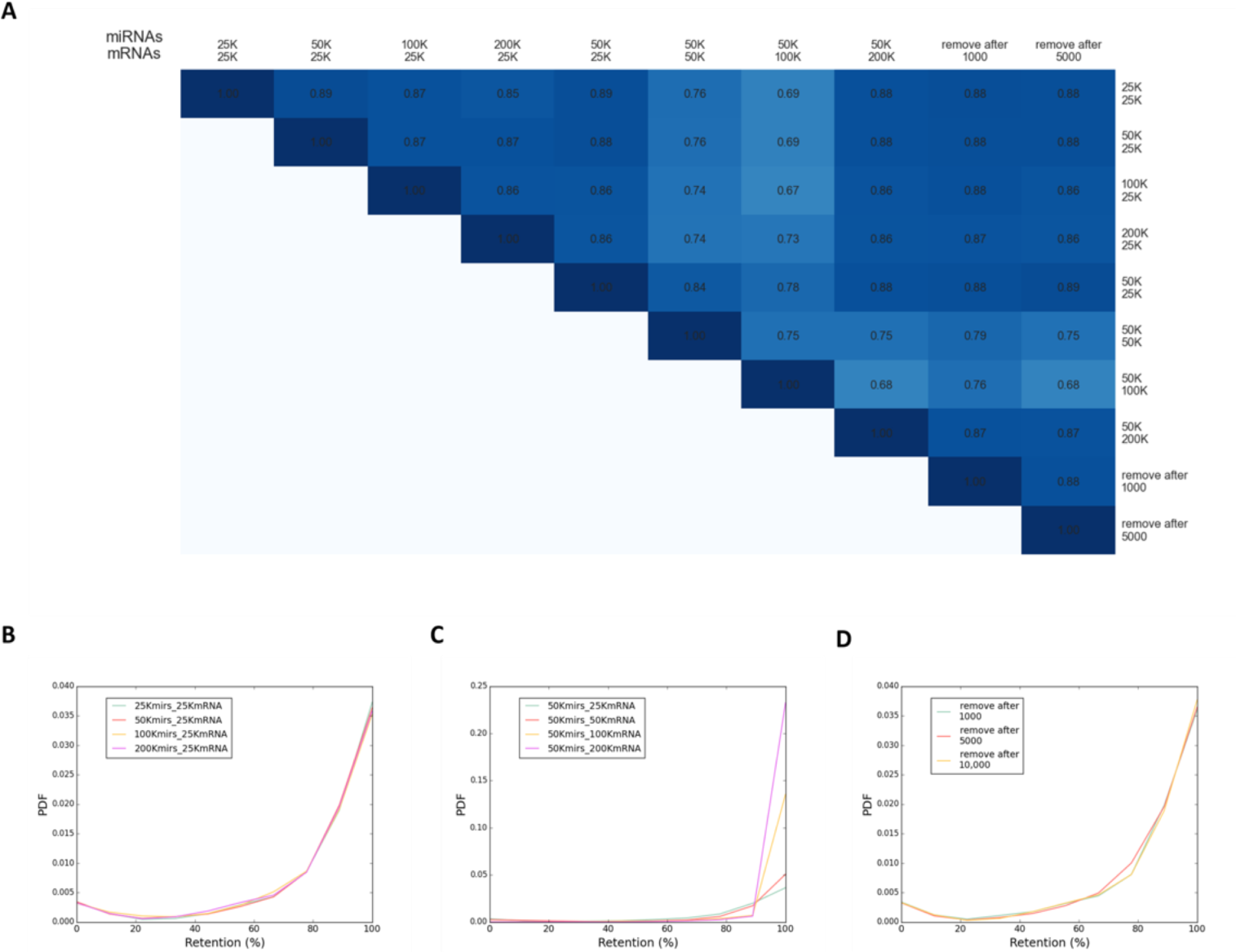

Supplement: S6 Fig — (A) Pearson correlation coefficients of the final retention after different simulation runs. Each run was conducted using a different set of parameters: different quantification and stoichiometry of miRNA mRNA ratio; different iteration interval between mRNA binding events, and varying the parameter for removal of a mRNA from the system. (B) The retention distribution at the end of different runs. The quantity of total mRNAs was fixed to 25k molecules, while its ratio with miRNAs quantity was varying to 1:1, 1:2, 1:4 and 1:8. (C) The retention distribution at the end of different runs. The quantity of total miRNAs was fixed to 50,000 molecules, while its ratio with mRNA quantity was set to 2:1, 1:1, 1:2 and 1:4. (D) The retention distribution at the end of different runs. The removal interval varied to 1000, 5000 and 10,000 iterations. (TIF) [file pcbi.1007204.s008.tif]

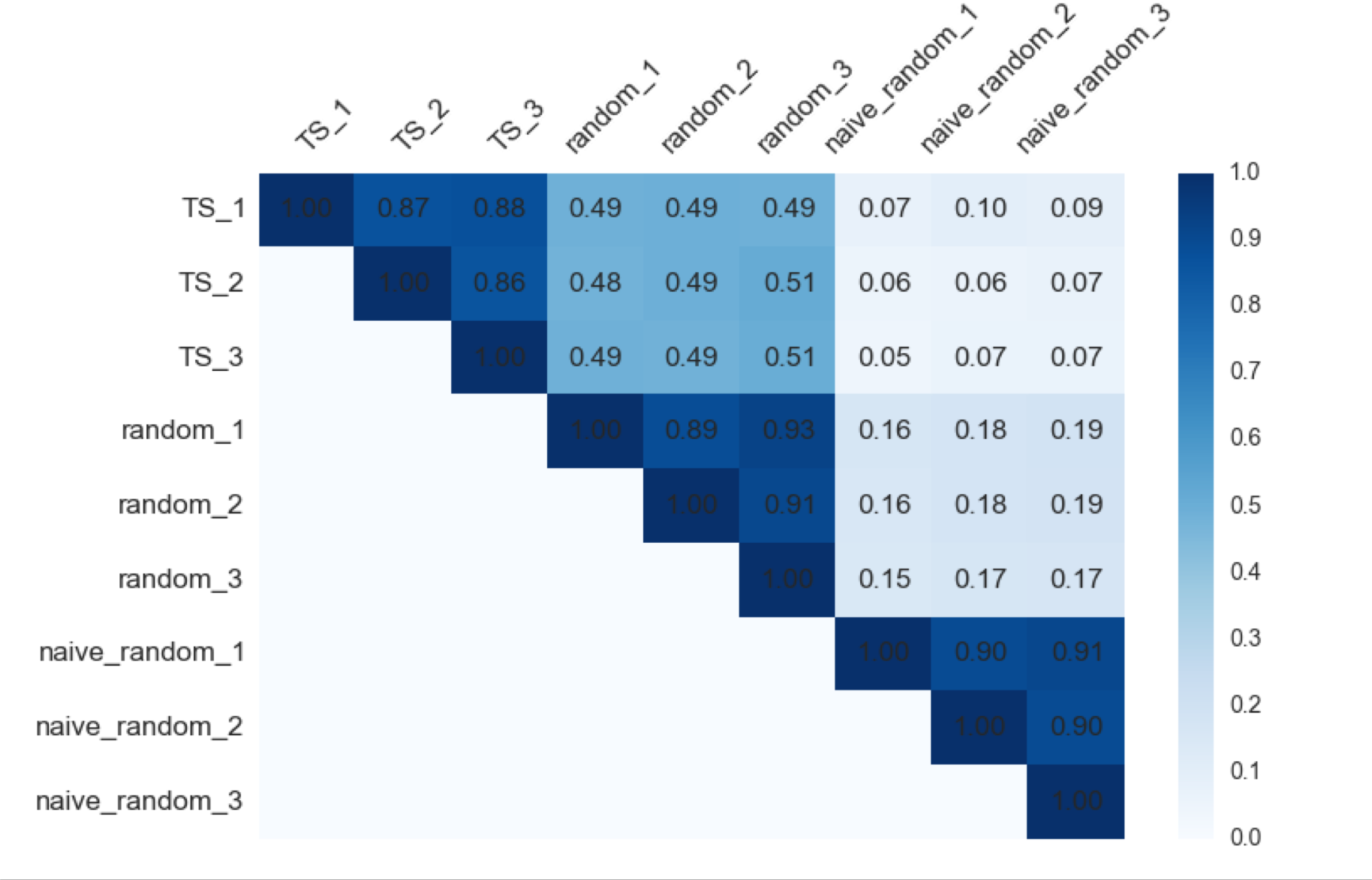

Supplement: S7 Fig — Pearson correlation of different simulation runs using three miRNA-mRNA interaction scoring tables: (i) TargetScan (marked as TS), (ii) randomized table of TargetScan. The randomization was done by fixing the total scores of each gene (row) and each miRNA (column) in the original TargetScan table. (iii) Naïve random table. In this case the original scores of TargetScan table were assigned to random pairing of miRNA–mRNA. Three independent simulation repetitions were performed using each of the above tables. Results for the Pearson rank correlations are color coded. Dark blue indicate correlation of 1. (TIF) [file pcbi.1007204.s009.tif]

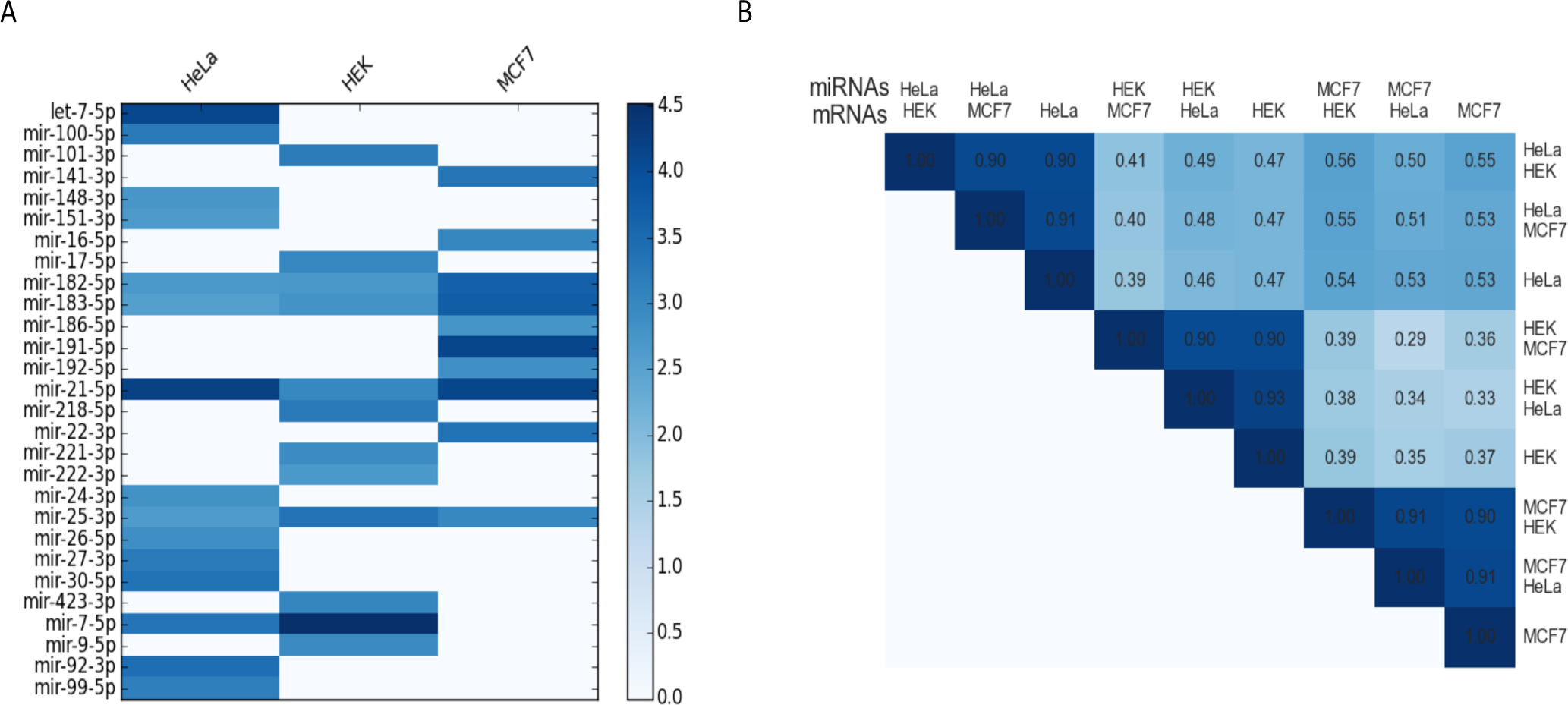

Supplement: S8 Fig — (A) Heatmap of miRNA from HeLa, HEK-293 and MCF-7 cell-lines in view of the abundance of each miRNA (colored in log10 scale) in each cell-type. The joint list of miRNAs includes the most abundant miRNAs that occupies 90% of the miRNA molecules (i.e. 45k out of 50k) in each cell-type. The fraction occupied by each of the listed miRNA, for each of the cell-type is available in S5 Dataset. (B) Pairs of miRNA and mRNA are shown according to their origin. Pearson correlation of the endpoint of the genes following 100k iterations and testing any of the genes that are above the minimal expression (5 molecules, 0.02% of total amount of mRNA). The number of mRNAs that are considered in the analyses are 516 for the pair of HeLa and HEK-293; 285 for the pair of HeLa and MCF-7 and 305 to the pair of HEK-293 and MCF-7. The p-value of the correlations are very significant for all pairs. All p-values correlation values are < 1e-15. (TIF) [file pcbi.1007204.s010.tif]
