# Supplementary material for: A cell-based probabilistic approach unveils the concerted action of miRNAs
Source: PLoS Comput Biol. 2019 Dec 2;15(12):e1007204. doi: 10.1371/journal.pcbi.1007204 (PMC6922470; doi:10.1371/journal.pcbi.1007204)
Supplement: S1 Text — It reports on the sensitivity and robustness with respect to numerous parameters’ changes. (DOCX) [file pcbi.1007204.s011.docx]

**S1 Text**: **COMICS design and parameters testing**

Supporting Information

**A.1. The design of COMICS: A probabilistic based miRNA-mRNA simulator**

The input for COMICS is the number of molecules for the expression profiles of miRNAs (total 50k molecules) and mRNAs (total 25k molecules) in the specific cell type, and a table of miRNA-mRNA interaction prediction extracted from TargetScan (see Materials and Methods). In addition, the simulator, supports a wide set of configurable parameters: (i) the number of total miRNA; (ii) the number of mRNA molecules in the cell; (iii) the number of iterations for completing the run; (iv) the number of iteration interval between miRNA-mRNA binding event and the mRNA removal; (v) a random removal of unbounded mRNAs according to predetermined decay rate of the mRNA as extracted and extrapolated from experimental data of mRNA half-life; (vi) addition of newly transcribed mRNAs during a configurable number of iterations interval; (vii) miRNAs or genes overexpression according to a selected multiplication factor for the degree of overexpression. (vii) incorporation of alternative miRNA-target mapping. It is also possible to activate the simulator by a set of random genes as an initial state of pre-existing iterations prior to the simulation run.

**A.2. COMICS sensitivity and robustness with respect to parameter changes**

We further tested the results of COMICS for reliably mimicking the competition of miRNAs in living cells, and the outcome on attenuation in mRNAs expression. This was performed by rigorous testing the sensitivity and robustness of the of results from the COMICS simulator following varying the operational parameters.

1. As the protocol we apply is probabilistic with a stochastic component for each iteration, we tested the variation of the simulator for independent runs. We compared the simulator output of three different runs using the same parameter set. We show a very high correlation (Pearson correlations are between 0.86 to 0.93, S6 Fig).
2. We tested the effect of the binding probability table. TargetScan score table (1) was shuffled using two randomization methods: (i) Constraint randomizing - the total score for each gene and for each miRNA was randomized while fixing the total probability values for each gene and each miRNA as presented in the original scoring table; (ii) Naïve randomization - In this case, we kept the interaction scores as provided by TargetScan matrix but randomized the MBS-miRNA pairs (see Methods). The correlation of the original TargetScan table and the randomized versions drops significantly. The Pearson correlations for the Constraint randomizing was 0.5, and there was no correlation at all using the table produced by the naïve randomization scheme (S6 Fig). This comparison shows the critical information that is provided by the prediction table for the output of the simulator.
3. We tested the effect of different initial quantities of miRNAs and mRNAs. Evidence of experimental data of total mRNA molecules from single cell estimates the number of transcripts to vary from 25k to 1M (2, 3) and the number of miRNAs vary dramatically with direct measurement of ~120k-200k molecules (4-6) in various cells, with limiting number of AGO proteins (at the range of 20k molecules). Those estimates greatly vary among cell types and developmental stages. The sensitivity of these absolute amounts to the expected effectiveness of the regulation was assessed experimentally and via a mathematical model (7). Results are shown in S5 Fig.
4. The effect of the difference in stoichiometry of miRNAs relative to mRNAs was tested by varying the absolute and relative ratio of the two sets of molecules. The final analyses are based on testing the final retention for 754, 755 and 694 expressed genes in HeLa, HEK-293 and MCF-7, respectively. cells after various simulation runs. We found very high and significant Pearson correlations between different sets of miRNA and mRNA quantities. In all experiments, the number of molecules in the cells were bounded by a minimum of 25k molecules for miRNA or mRNAs. The default setting was 50k and 25k molecules for miRNA and mRNA respectively. The ratio between miRNAs and mRNAs was tested from 1:1 to 8:1 ratio. Similarly, the ratio of mRNA to miRNA was elevated from the default value of 0.5:1 to 2:1. In all instances, the effect on the endpoint was minimal and a change in ratio of up to 8 folds had an effect mostly on the kinetics rather than on the endpoint of simulation. The notion of miRNA regulating the gene expression by thresholds rather than by simple buffering was proposed (5). Changing the cell miRNA concentration without affecting the stoichiometry of 2:1 resulted in a minor change in the decay rate. Changes in miRNA quantity shows no significant changes in the final retention distribution (KS test p-values range between 0.80 to 1.0, Results are shown in S5 Fig.
5. Testing the sensitivity of the system to different expression profile as input was achieved by comparing the performance of the simulation output for three different cell-types. The simulation was done on HeLa, HEK-293 and MCF-7 expression profiles. We found a significant difference in all expressed genes (KS p-values ranges between 0.07 to 3.38e-38). Results are shown in S7 Fig.
6. The next parameter we tested is the interval of iteration between the pairing of miRNA-mRNA and the removal of the mRNA (mimicking the gene expression attenuation by cleavage by AGO-2). We change the setting of the mRNA elimination interval by changing the parameter from 1000, 5000 and 10,000 iterations (i.e. each 1%, 5% and 10% of the simulation run). We found no significant changes in the simulation results using any of those values (S5 Fig).
7. The sampling of miRNAs and mRNA and assessing their pairing is done according to a table of probability scores that represents a rich computational-experimental body of knowledge (1). Once two molecules are successfully paired, our tables of probabilities get appropriately updated (Fig 3A). We show that the mRNA retention statistics at the endpoint of the simulation runs of COMICS is surprisingly robust under a wide range of parameters. From computational considerations and based on numerous empiric observations, each pair of miRNA-target is assigned a score that reflects the calculated degree of downregulation (e.g., (8). Correlating the profile of overexpressed miRNAs from in-vitro experiments with the outcome of downregulated genes allows a refinement of the miRNA-target prediction scores (9, 10), and inferring a probabilistic measure for the effectiveness for each prediction. Along with this line, TargetScan prediction tool reports on the probability of a given MBS and its combination to effectively interact with the miRNA at hand (1). We show that the values calculated in the TargetScan interaction table (1) are the most sensitive parameters for the implementation of COMICS (Supplemental S6 Fig). If we run the simulation after the MBS interaction values undergo a constrained randomization modification, then our self-correlations drop drastically. In all our simulations we use the same probabilities table.
8. A slight change in the definition for the sensitive and stable genes have a minimal impact on the results. Specifically, an identical gene list resulted for the cross-miRNA sensitive genes where the threshold for a retention level was reduced to <20% (instead of <50%). However, changing the definition for cross-miRNA stable genes (e.g. high retention >80% for ≥90% of the tested miRNAs) slightly increased the gene list. In both cases, the downstream functional analysis was insensitive to these slight changes in definitions.

**References**

1. Agarwal V, Bell GW, Nam JW, Bartel DP. Predicting effective microRNA target sites in mammalian mRNAs. Elife. 2015;4.

2. Marinov GK, Williams BA, McCue K, Schroth GP, Gertz J, Myers RM, et al. From single-cell to cell-pool transcriptomes: stochasticity in gene expression and RNA splicing. Genome Res. 2014;24(3):496-510.

3. Ramskold D, Luo S, Wang YC, Li R, Deng Q, Faridani OR, et al. Full-length mRNA-Seq from single-cell levels of RNA and individual circulating tumor cells. Nat Biotechnol. 2012;30(8):777-82.

4. Janas MM, Wang B, Harris AS, Aguiar M, Shaffer JM, Subrahmanyam YV, et al. Alternative RISC assembly: binding and repression of microRNA-mRNA duplexes by human Ago proteins. RNA. 2012;18(11):2041-55.

5. Mukherji S, Ebert MS, Zheng GX, Tsang JS, Sharp PA, van Oudenaarden A. MicroRNAs can generate thresholds in target gene expression. Nature genetics. 2011;43(9):854-9.

6. Denzler R, Agarwal V, Stefano J, Bartel DP, Stoffel M. Assessing the ceRNA hypothesis with quantitative measurements of miRNA and target abundance. Molecular cell. 2014;54(5):766-76.

7. Bosson AD, Zamudio JR, Sharp PA. Endogenous miRNA and target concentrations determine susceptibility to potential ceRNA competition. Molecular cell. 2014;56(3):347-59.

8. Betel D, Koppal A, Agius P, Sander C, Leslie C. Comprehensive modeling of microRNA targets predicts functional non-conserved and non-canonical sites. Genome Biol. 2010;11(8):R90.

9. Li Y, Liang C, Wong KC, Jin K, Zhang Z. Inferring probabilistic miRNA-mRNA interaction signatures in cancers: a role-switch approach. Nucleic Acids Res. 2014;42(9):e76.

10. Bloom RJ, Winkler SM, Smolke CD. A quantitative framework for the forward design of synthetic miRNA circuits. Nat Methods. 2014;11(11):1147-53.
